# Supplementary material for: A native bacterial consortium degrades estriol in domestic sewage and activated sludge via the 4,5-seco pathway and requires estriol to retain its biodegradation phenotype
Source: Microbiol Spectr. 2025 Aug 26;13(10):e00741-25. doi: 10.1128/spectrum.00741-25 (PMC12502722; doi:10.1128/spectrum.00741-25)
Supplement: Supplemental figures and table — Fig. S1 to S32 and Table S1. [file spectrum.00741-25-s0001.pdf]

Supplementary Material

Microbiology Spectrum

A native bacterial consortium degrades estriol in domestic sewage and activated sludge via the 4,5-*seco* pathway and requires estriol to retain its biodegradation phenotype

Jaleela S. Hashem[1], Wael Ismail[2*], Yin-Ru Chiang[3], Vartul Sangal[4], Dorra Hentati[2#], Nasser Abotalib[2], Adnan A. Bekhit[1,5]

[1]Allied Health Sciences Department, College of Health and Sport Sciences, University of Bahrain, P.O. Box 32038, Bahrain

[2]Centre of Environmental and Biological Studies, Arabian Gulf University, Bahrain

[3]Biodiversity Research Center, Academia Sinica, Taipei, Taiwan

[4]Faculty of Health and Life Sciences, Northumbria University, Newcastle upon Tyne, United Kingdom

[5]Department of Pharmaceutical Chemistry, Faculty of Pharmacy, Alexandria University, Alexandria 21521, Egypt

[*]Corresponding author: Tel: +973-36146948; e-mail: waelame@agu.edu.bh

[#] Current Affiliation: Department of Food, Environmental and Nutritional Sciences (DeFENS)

University of Milan via Celoria 2, 20133 Milano, Italy

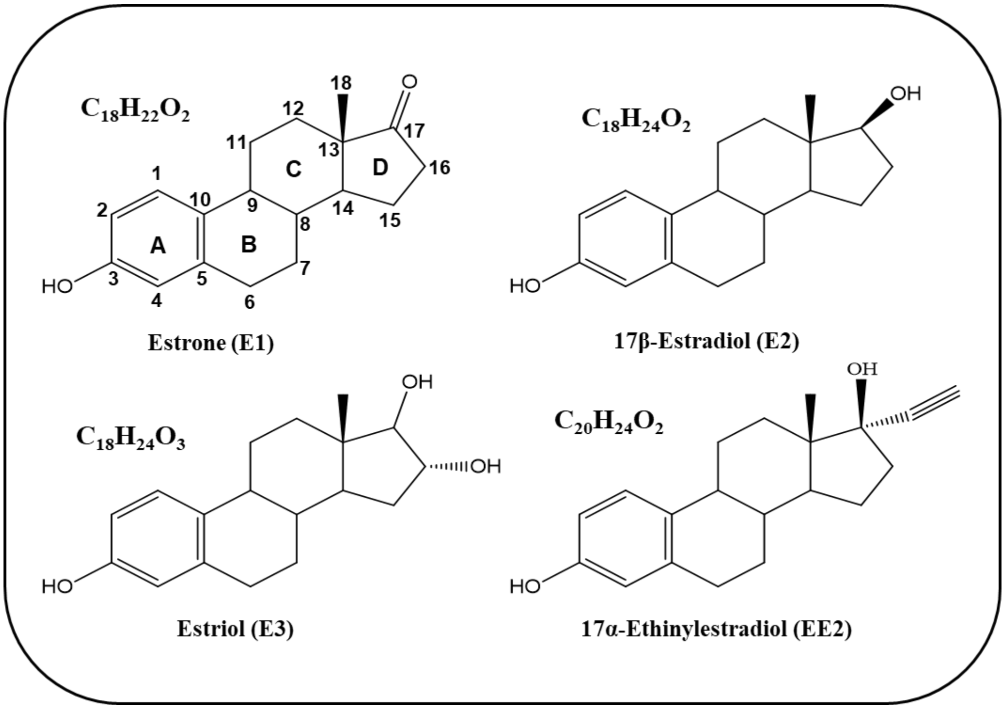

Fig. S1: Chemical structures of steroid estrogens.

a b

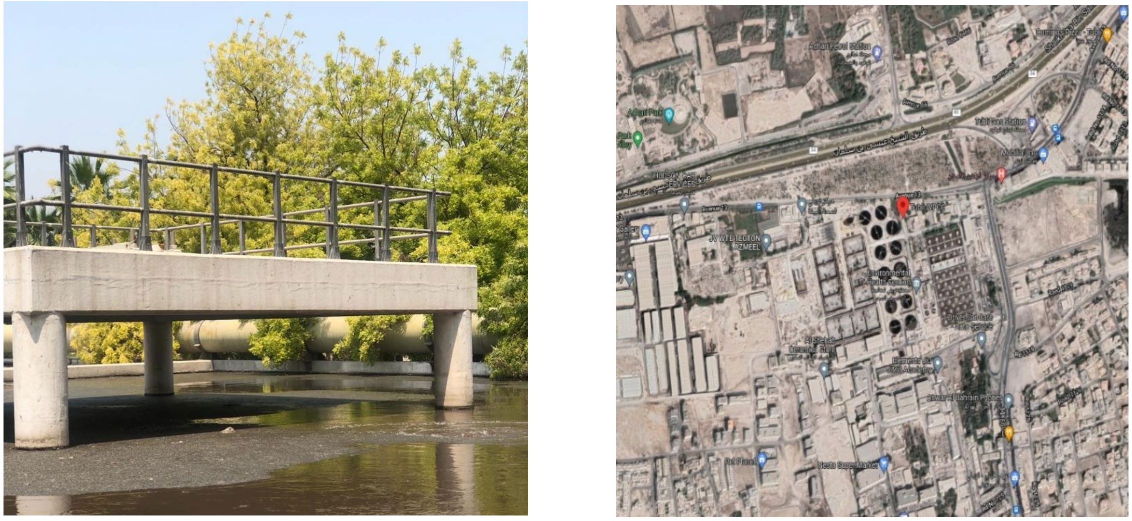

Fig. S2: Tubli wastewater treatment plant. (a) an aeration tank, (b) a top view of the plant and the clarifiers.

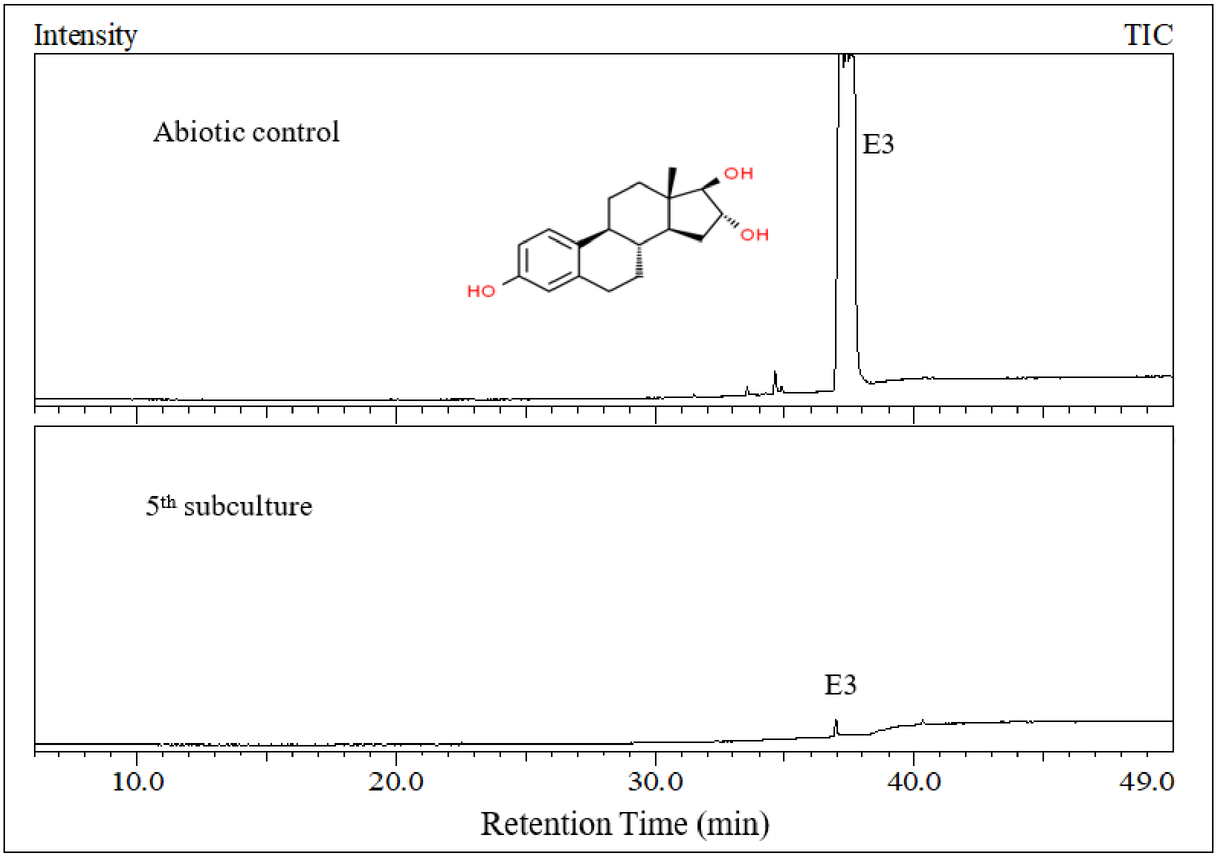

Fig. S3: GC-MS analysis showing total ion chromatograms of the 5th enrichment subculture of inflow wastewater samples containing 1 mM E3. Culture samples were extracted after 4 days of incubation and analyzed by GC-MS. E3 was added from a stock solution in DMSO. All cultures were in 100 mL CDM and were incubated at 30 °C in an orbital shaker (180 rpm).

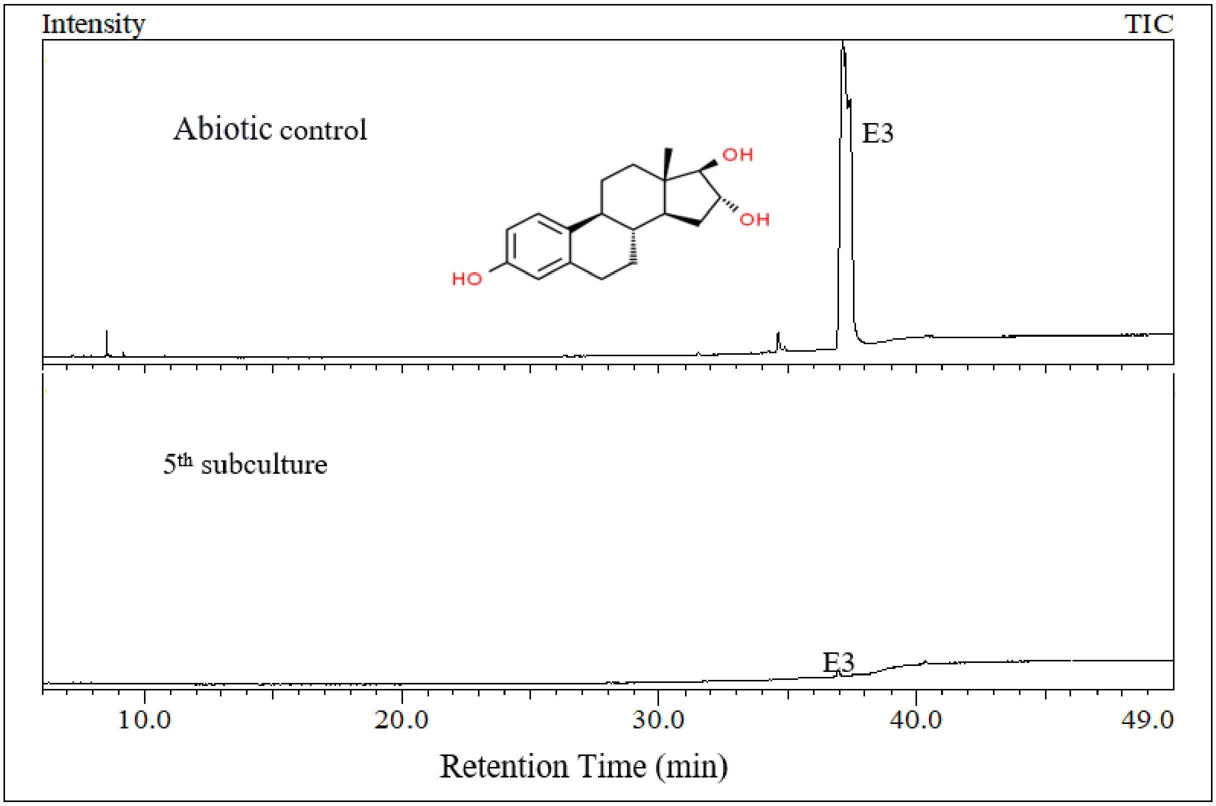

Fig. S4: GC-MS analysis showing total ion chromatograms of the 5th enrichment subculture of thickened sludge samples containing 1 mM E3. Culture samples were extracted after 4 days of incubation and analyzed by GC-MS. E3 was added from a stock solution in DMSO. All cultures were in 100 mL CDM and were incubated at 30 °C in an orbital shaker (180 rpm).

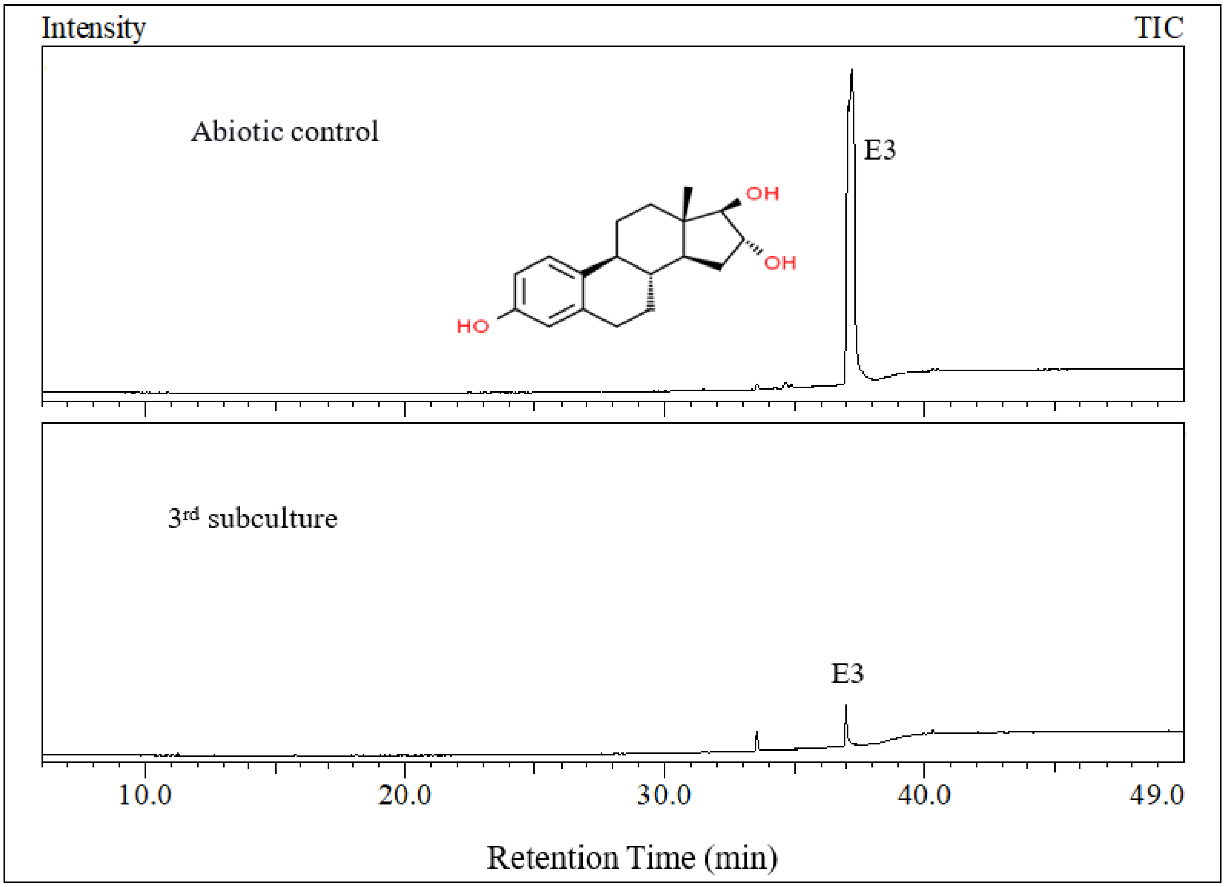

Fig. S5: GC-MS analysis showing total ion chromatograms the 3rd enrichment subculture of activated sludge samples containing 1 mM E3. Culture samples were extracted after 4 days of incubation and analyzed by GC-MS. E3 was added from a stock solution in DMSO. All cultures were in 100 mL CDM and were incubated at 30 °C in an orbital shaker (180 rpm).

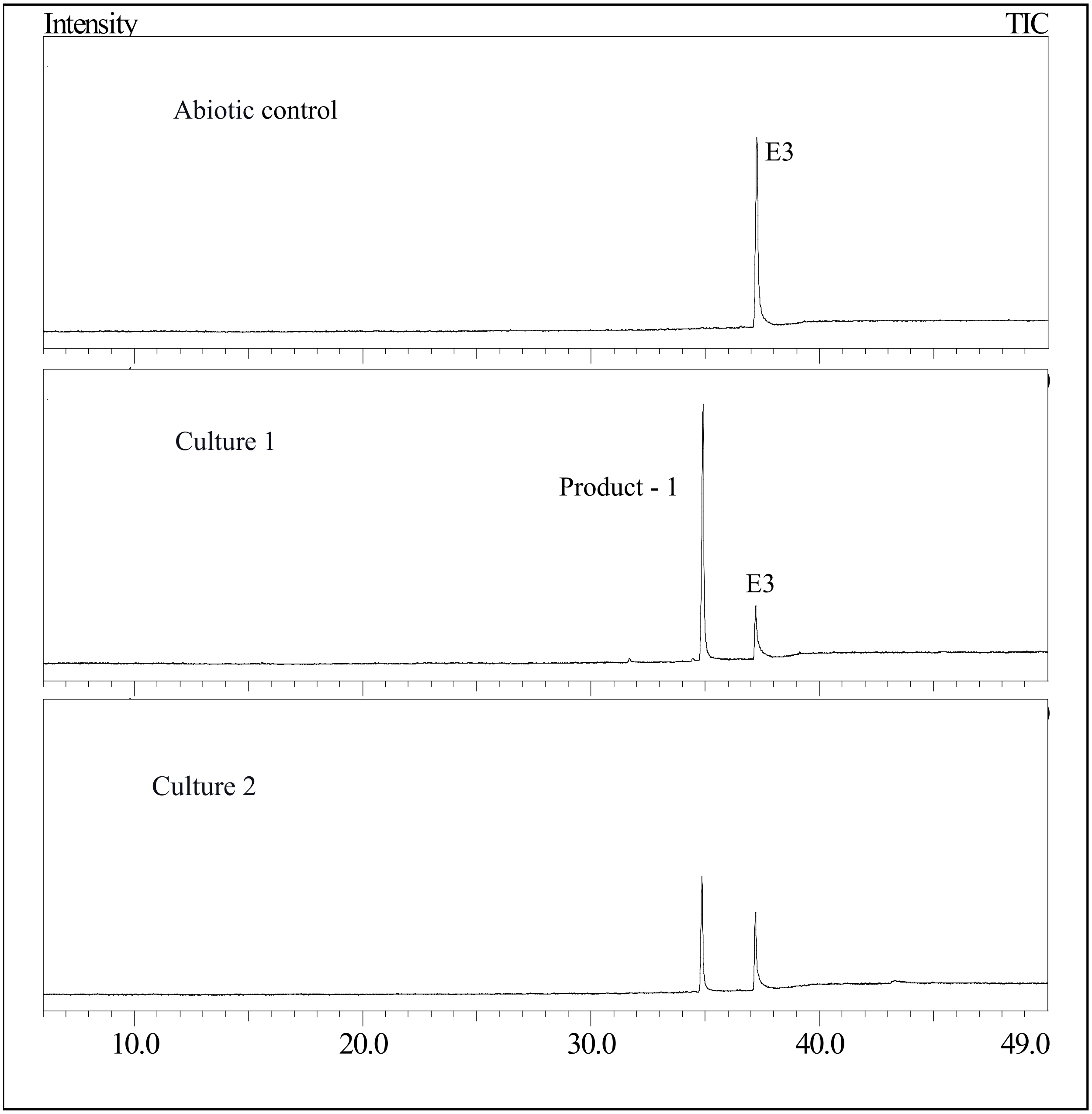

Fig. S6: Total ion chromatograms from GC-MS analysis showing degradation of E3 by cultures of the thickened sludge consortium in CDM containing 0.1 mM E3, which was added from a DMSO stock solution. Results from two biological replicates are shown. Culture samples were retrieved after 5 days of incubation, extracted with ethylacetate and analyzed by GC-MS.

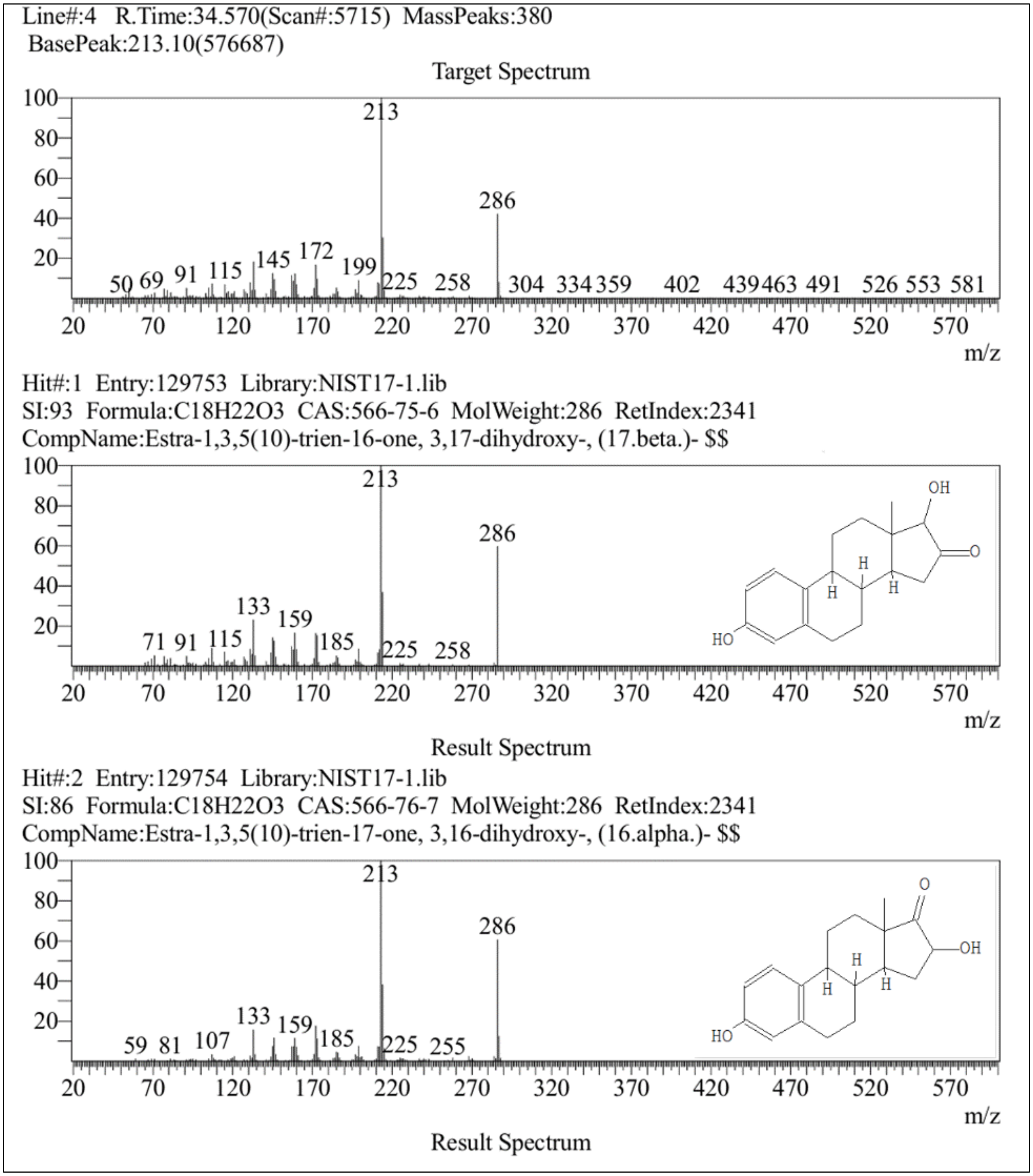

Fig. S7: Legend on next page

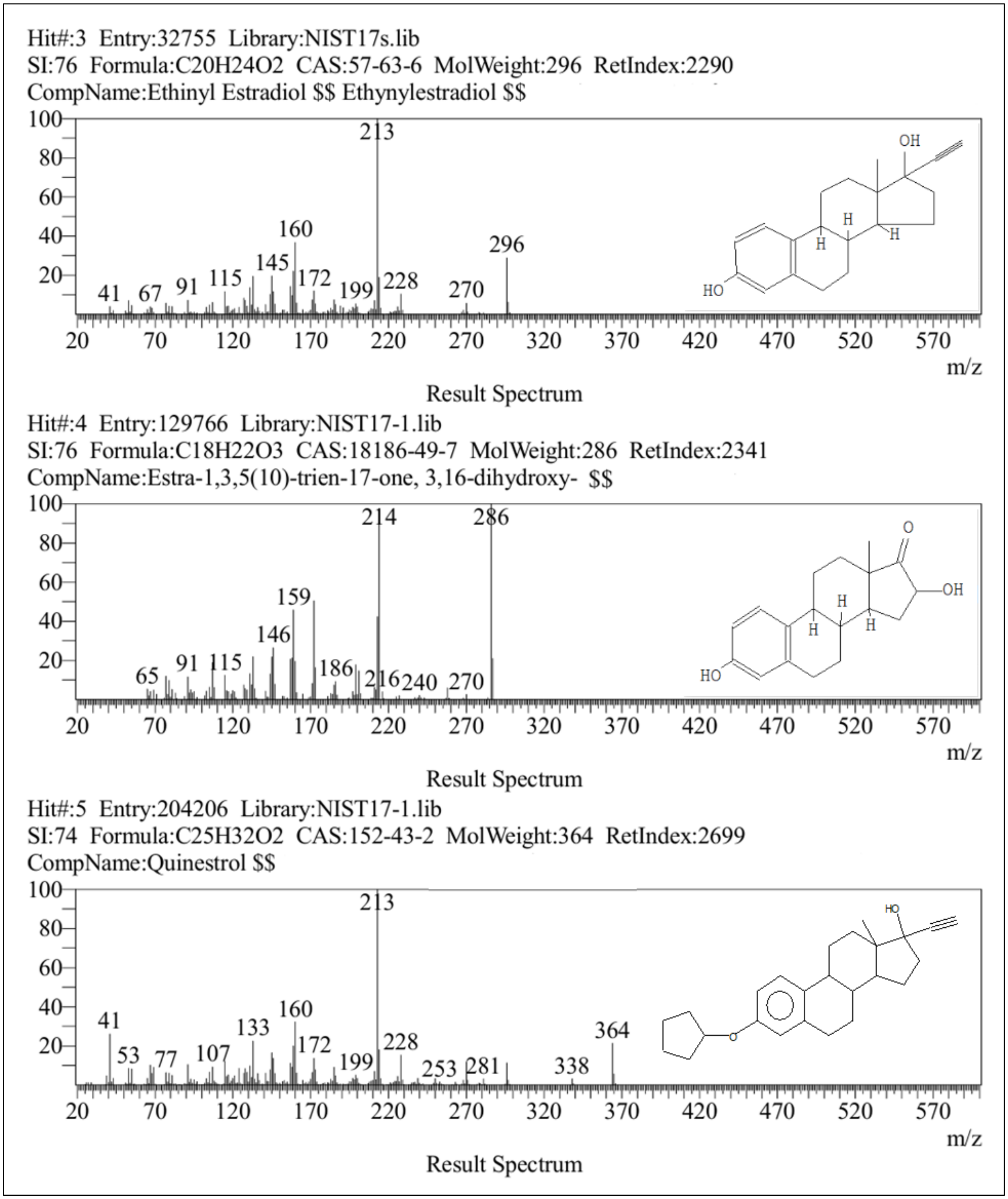

Fig. S7: Predicted mass spectrum of and product-1 and the matching spectra from the GC-MS spectral library NIST17-1.

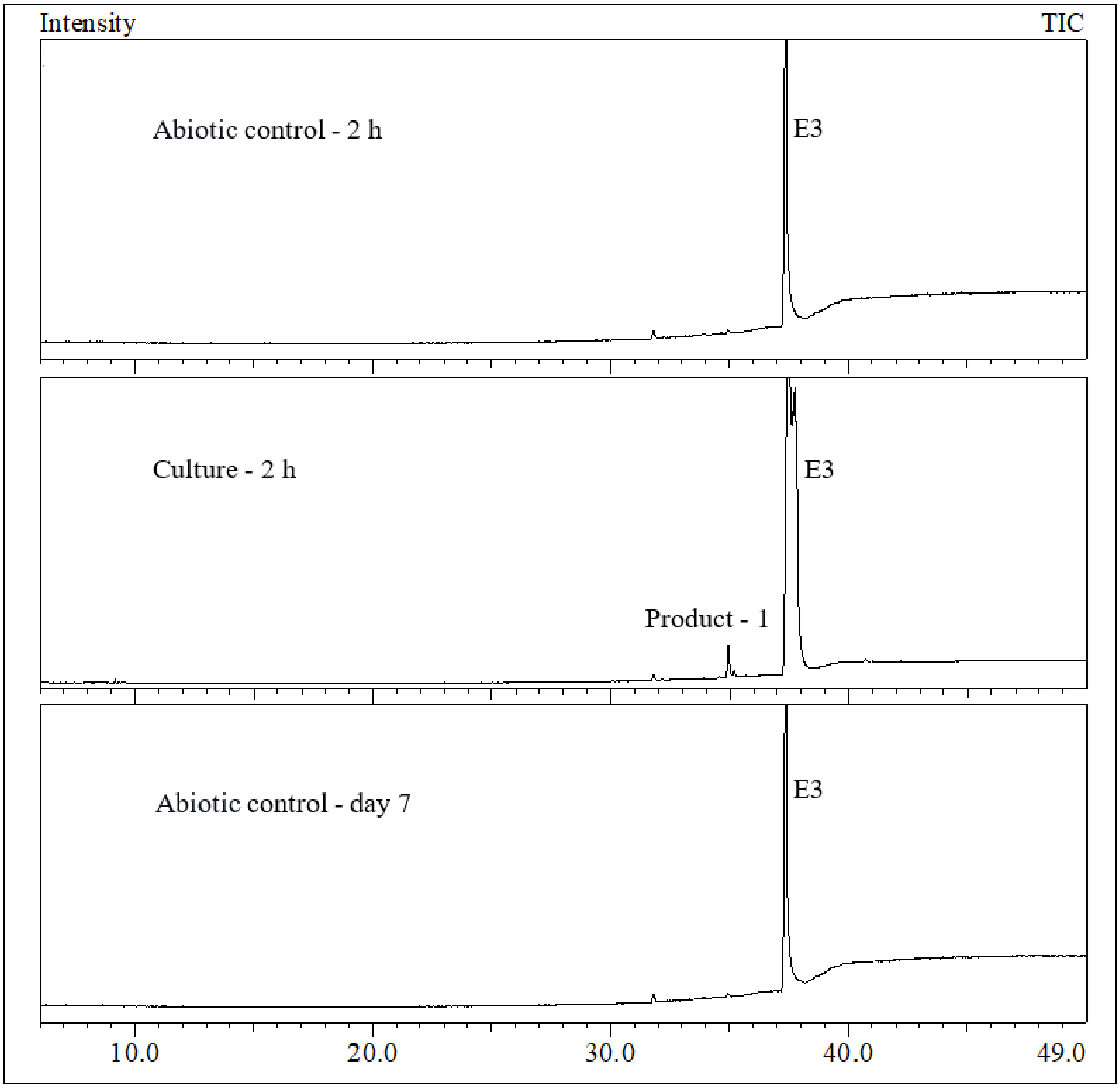

Fig. S8: Legend on next page

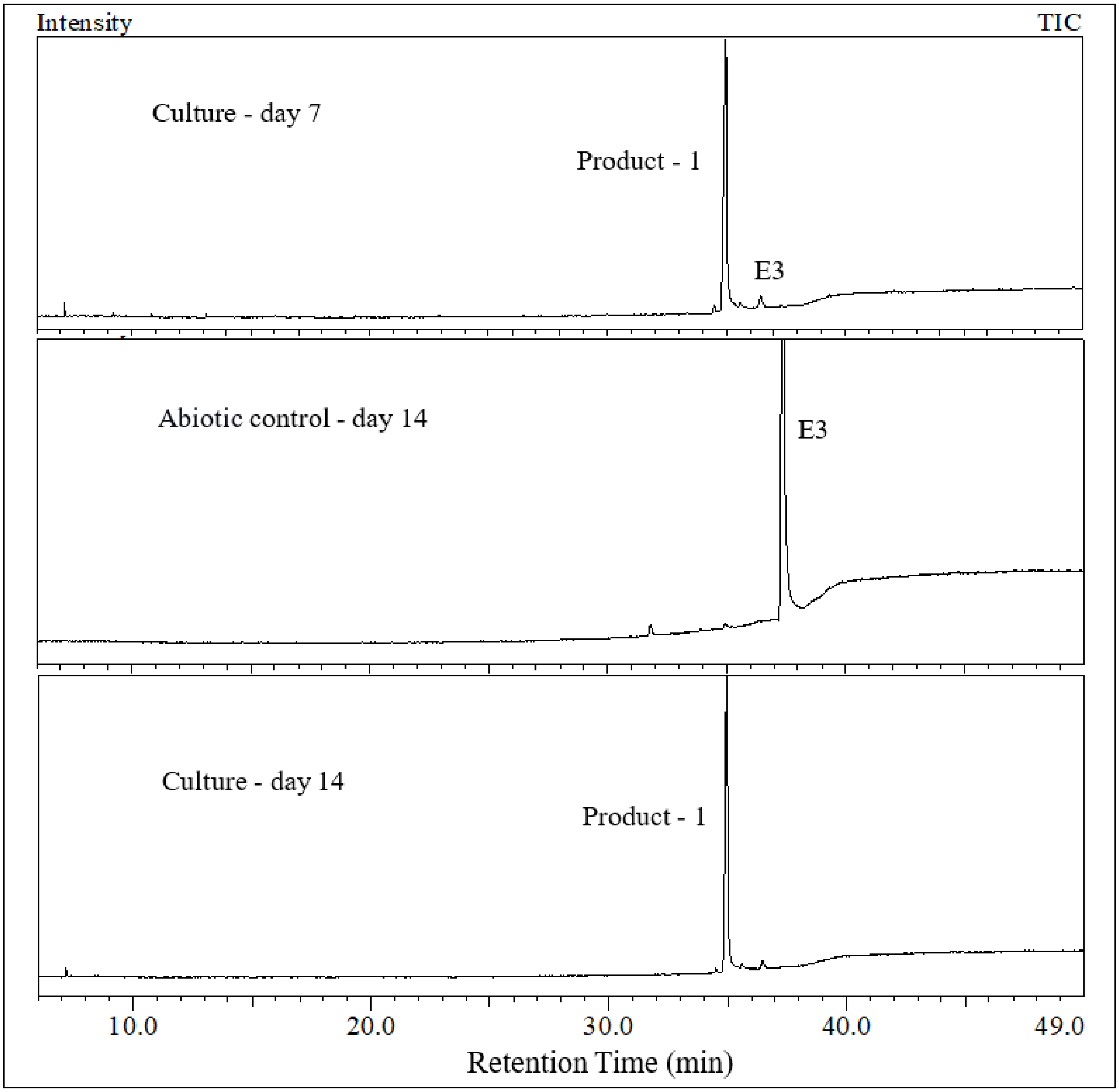

Fig. S8: Total ion chromatograms from GC-MS analysis showing transformation of E3 to unknown products by strain TH-11 cultures grown in CDM containing 0.1 mM E3 and incubated for different time intervals. E3 was added from a stock solution in DMSO. All cultures were in 100 mL CDM and were incubated at 30 °C in an orbital shaker (180 rpm).

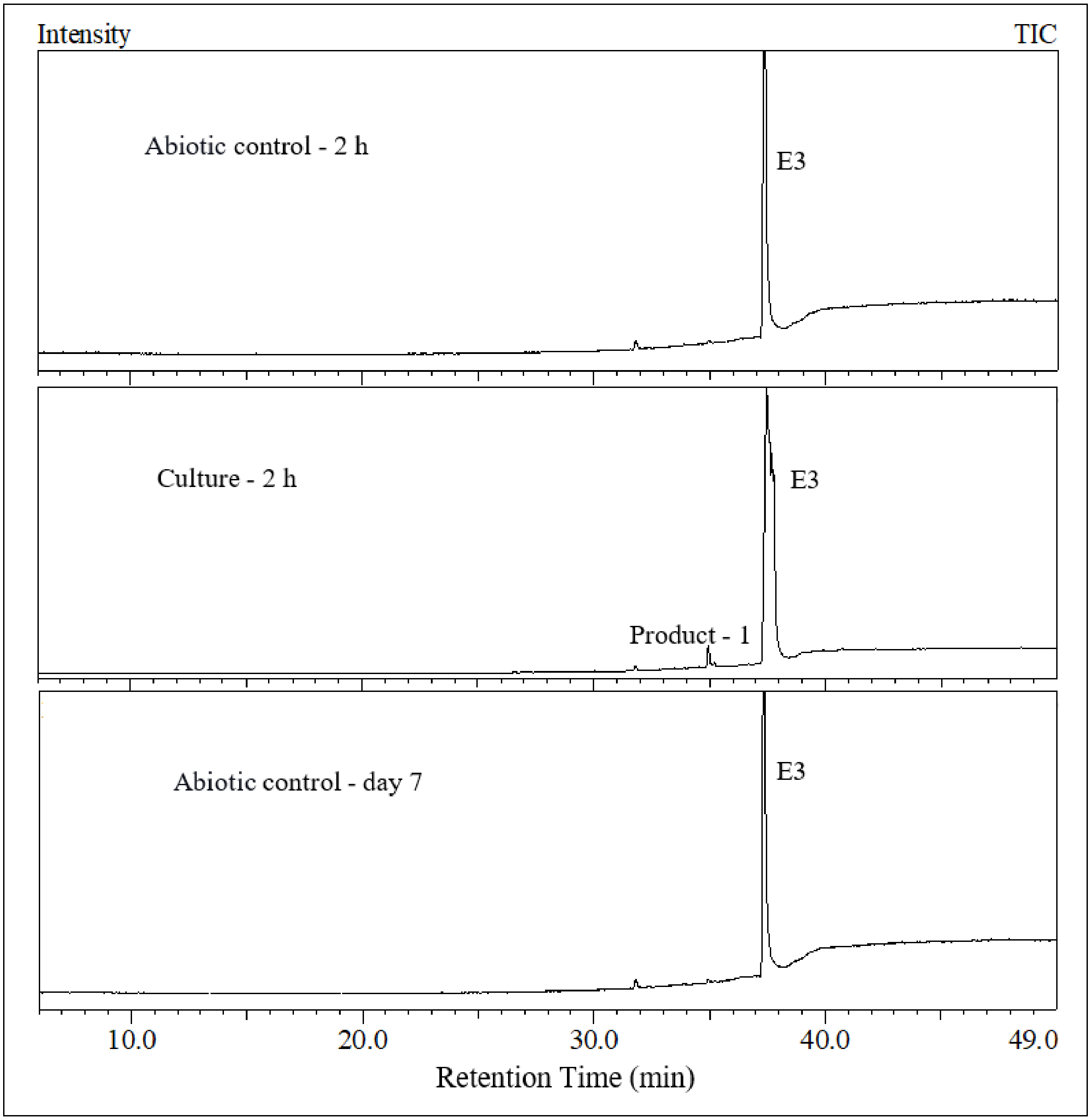

Fig. S9: Legend on next page

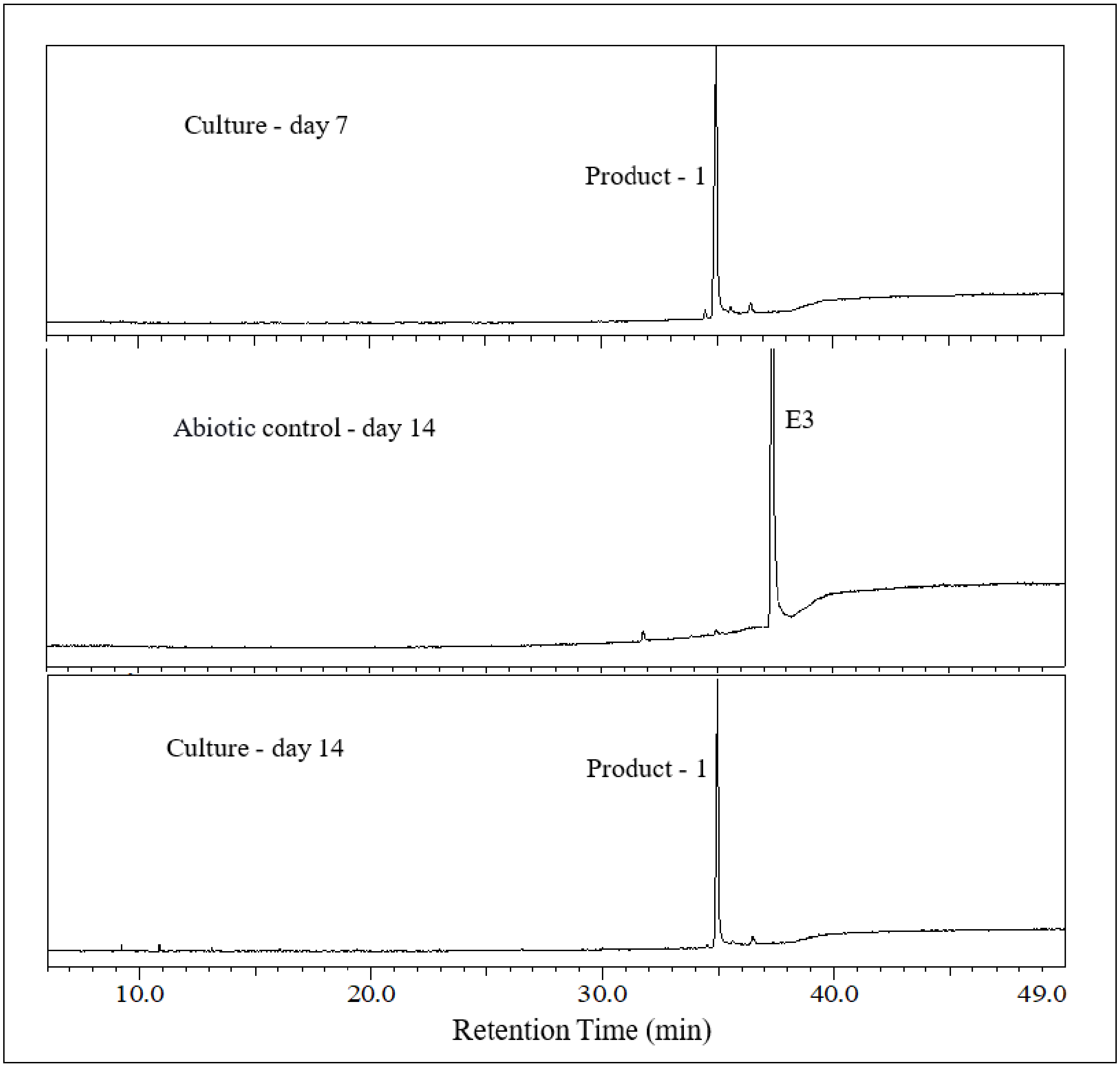

Fig. S9: Total ion chromatograms from GC-MS analysis showing transformation of E3 to unknown products by strain TH-16 cultures grown in CDM containing 0.1 mM E3 and incubated for different time intervals. E3 was added from a stock solution in DMSO. All cultures were in 100 mL CDM and were incubated at 30 °C in an orbital shaker (180 rpm).

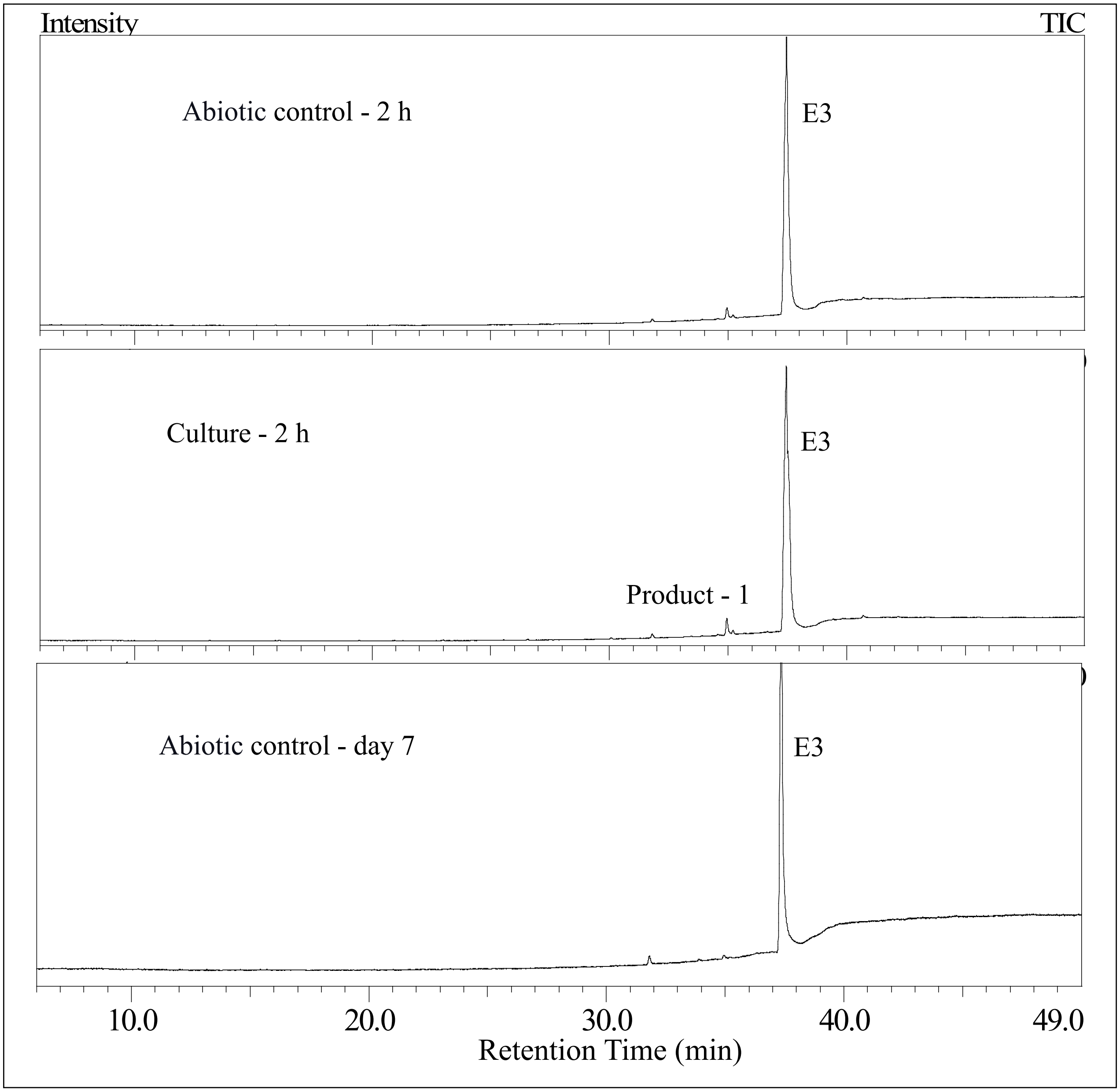

Fig. S10: Legend on next page

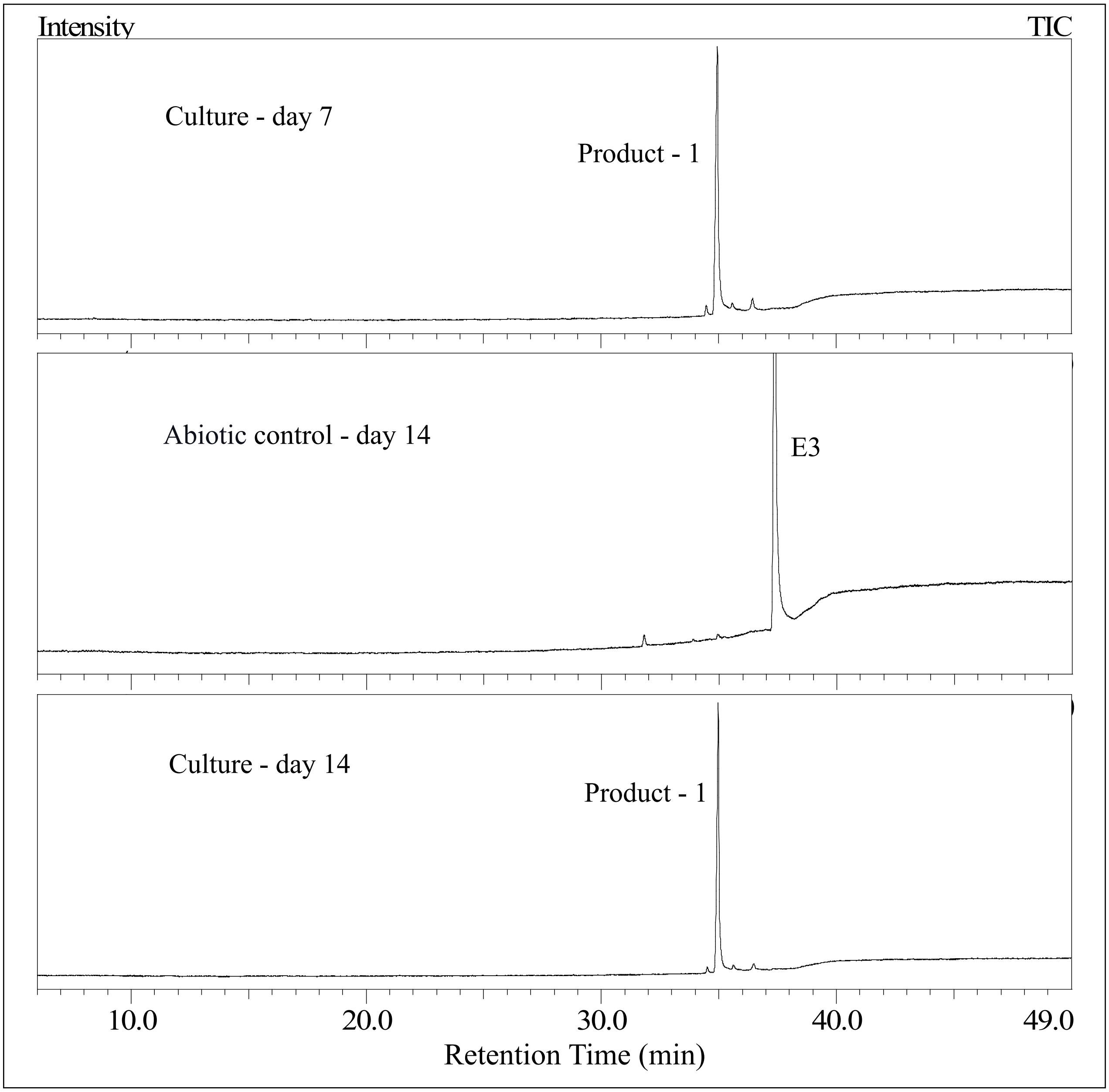

Fig. S10: Total ion chromatograms from GC-MS analysis showing transformation of E3 to unknown products by strain TH-30 cultures grown in CDM containing 0.1 mM E3 and incubated for different time intervals. E3 was added from a stock solution in DMSO. All cultures were in 100 mL CDM and were incubated at 30 °C in an orbital shaker (180 rpm).

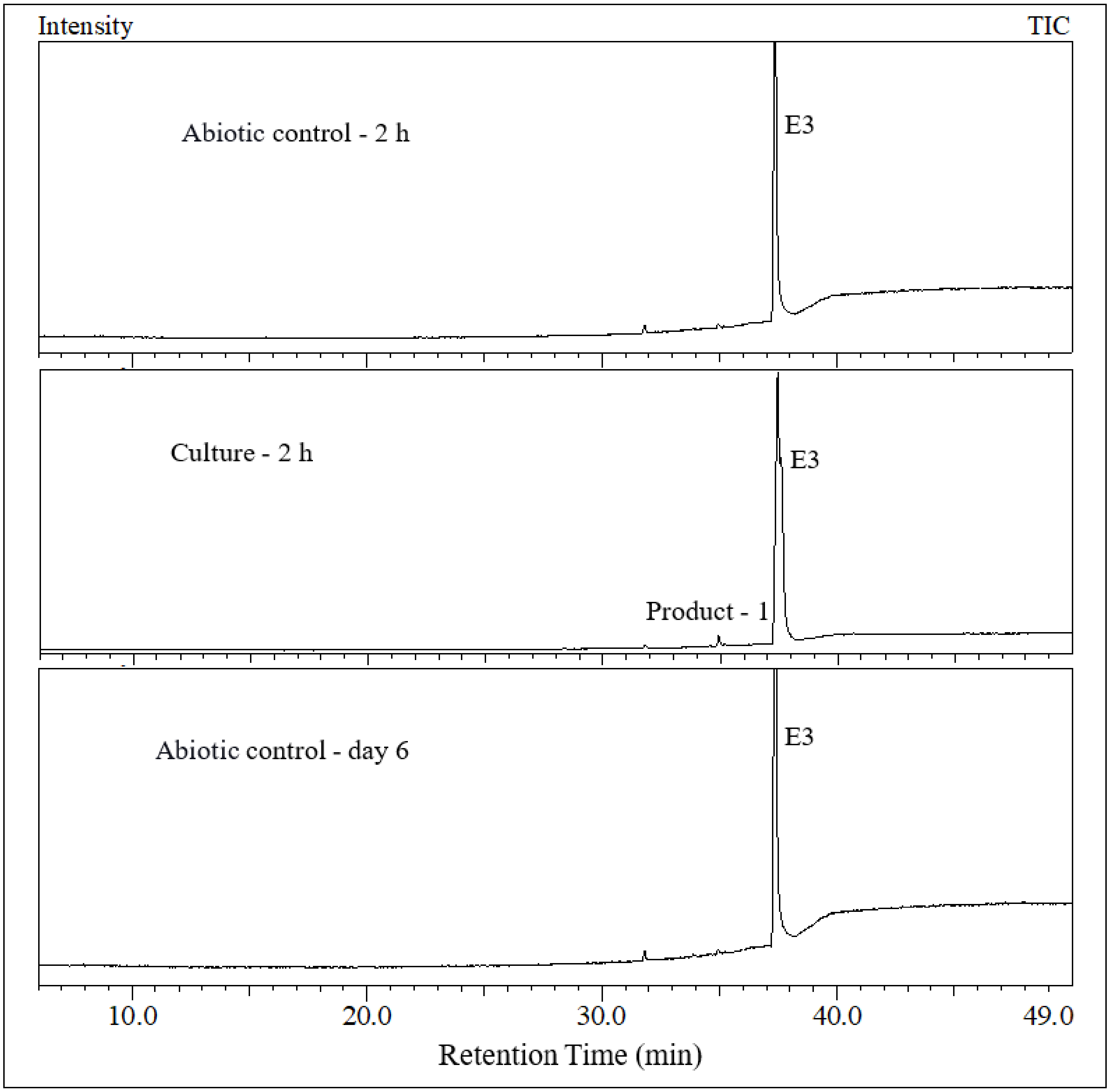

Fig. S11: Legend on next page

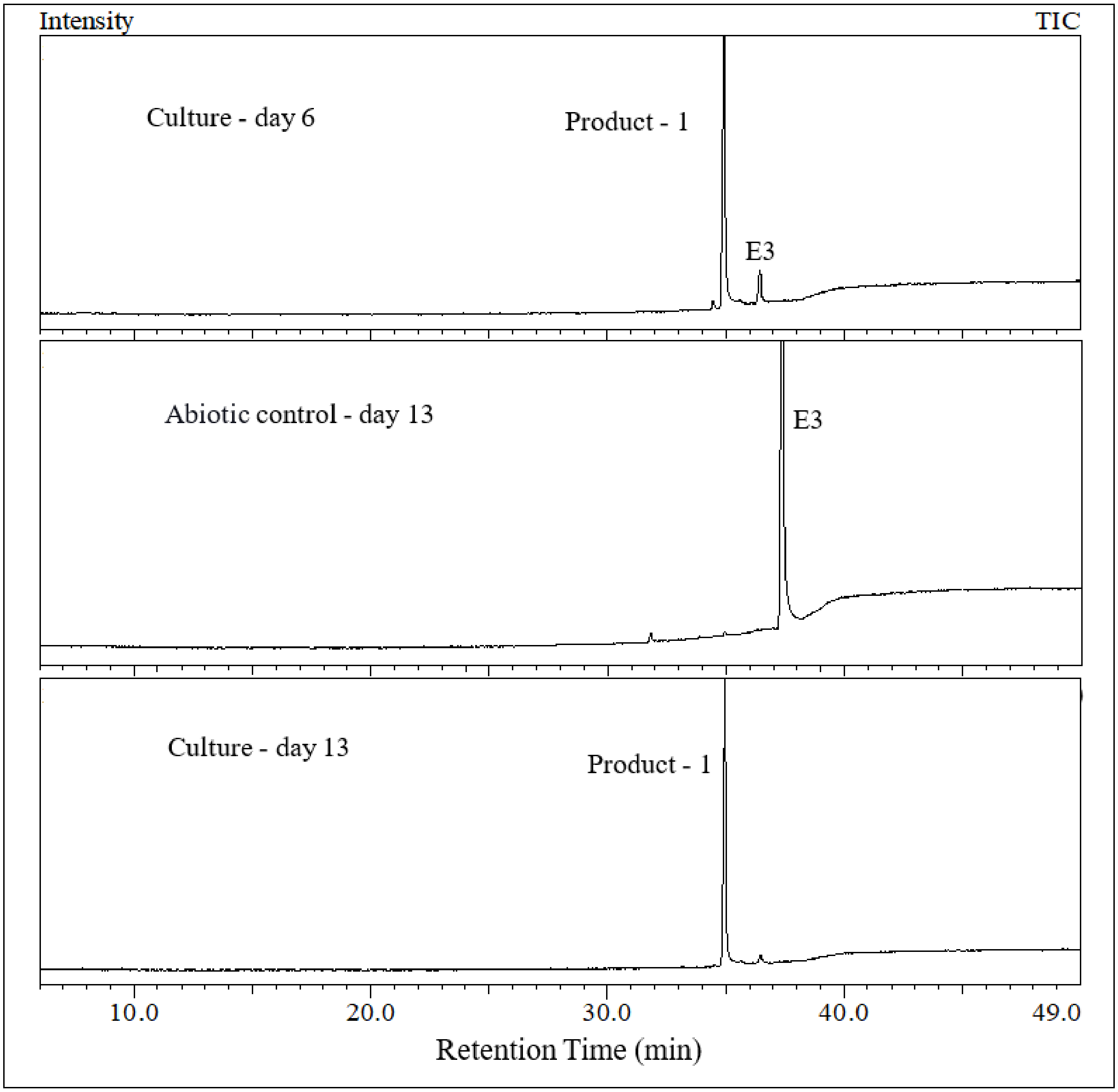

Fig. S11: Total ion chromatograms from GC-MS analysis showing transformation of E3 to unknown products by strain IF-20 cultures grown in CDM containing 0.1 mM E3 and incubated for different time intervals. E3 was added from a stock solution in DMSO. All cultures were in 100 mL CDM and were incubated at 30 °C in an orbital shaker (180 rpm).

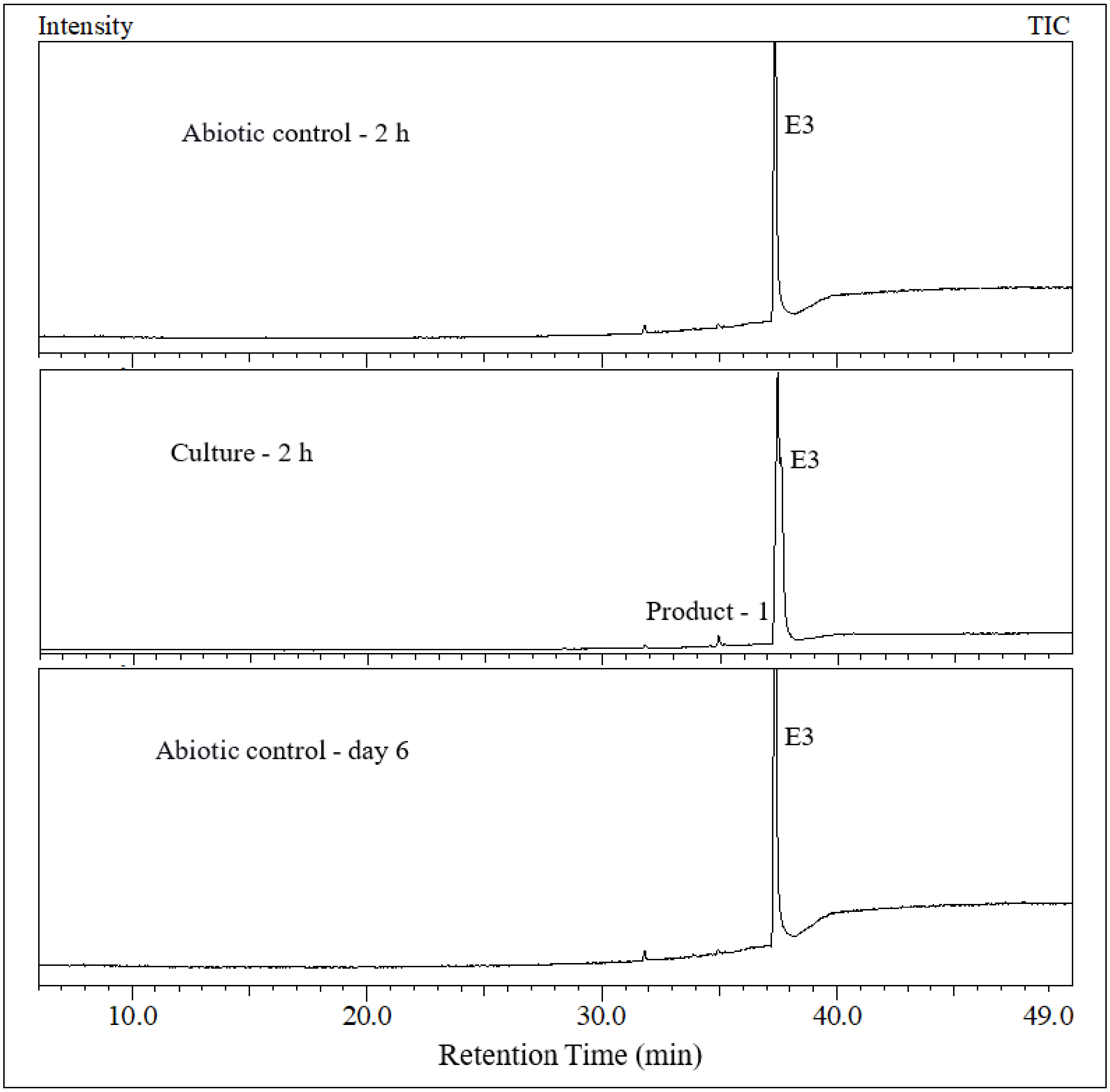

Fig. S12: Legend on next page

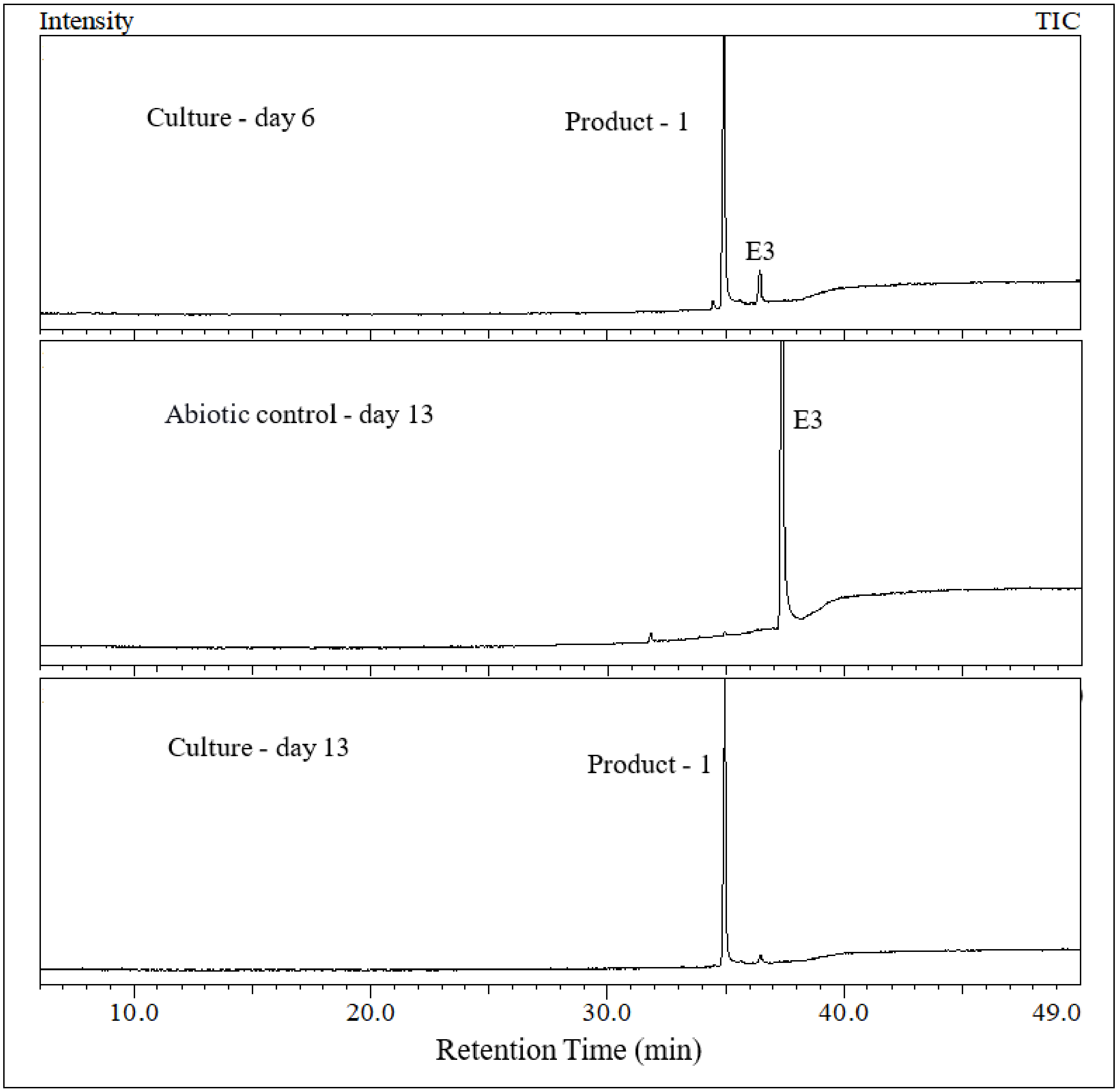

Fig. S12: Total ion chromatograms from GC-MS analysis showing transformation of E3 to unknown products by strain AS-31 cultures grown in CDM containing 0.1 mM E3 and incubated for different time intervals. E3 was added from a stock solution in DMSO. All cultures were in 100 mL CDM and were incubated at 30 °C in an orbital shaker (180 rpm).

A

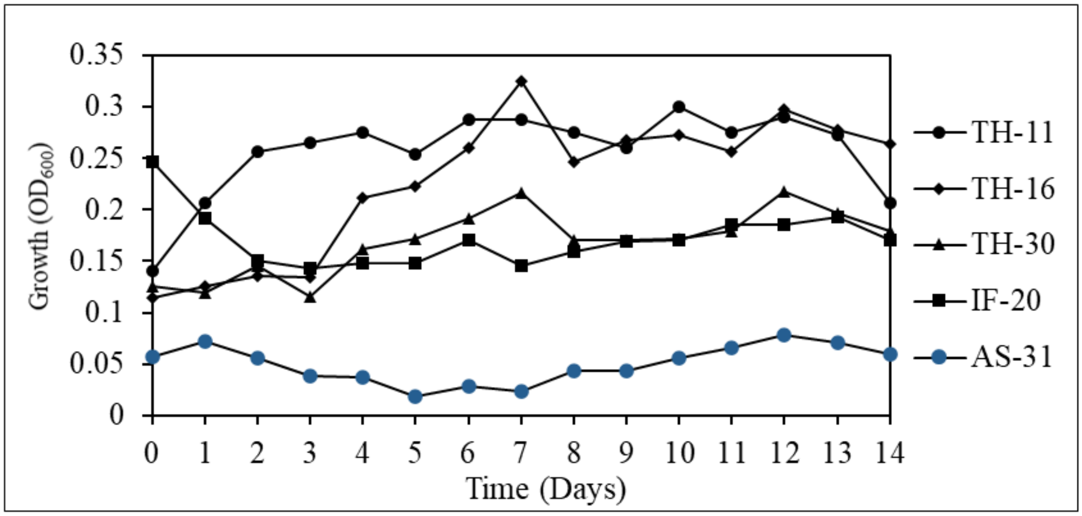

B

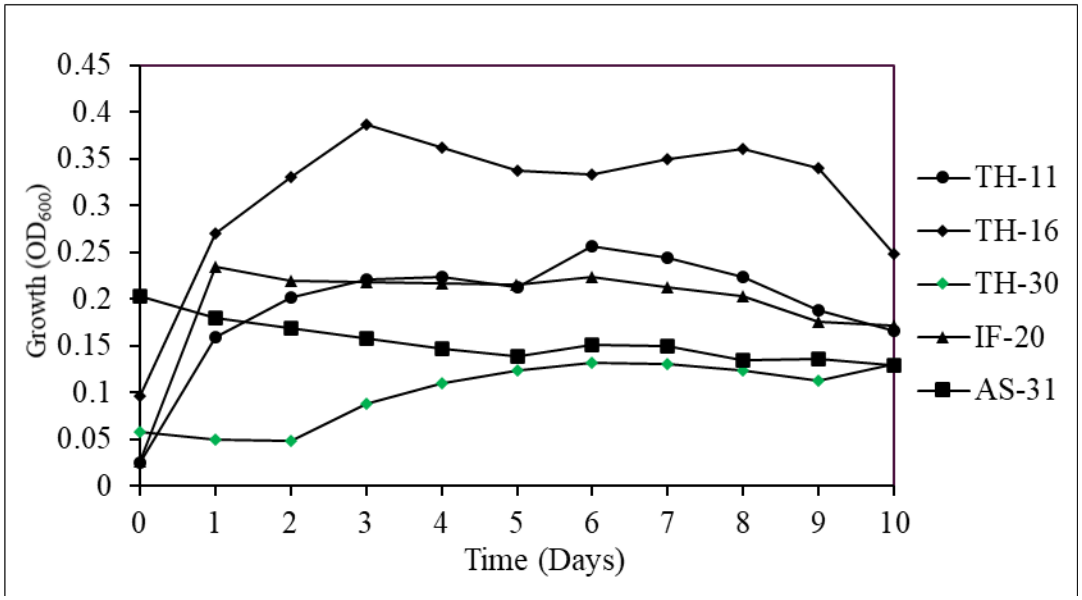

Fig. S13: Growth profiles of the isolated strains in CDM containing either 0.1 mM E3 (dissolved in DMSO) (A) or 50 mg/L E3 crystals (B). All cultures were in 100 mL CDM and were incubated at 30 °C in an orbital shaker (180 rpm).

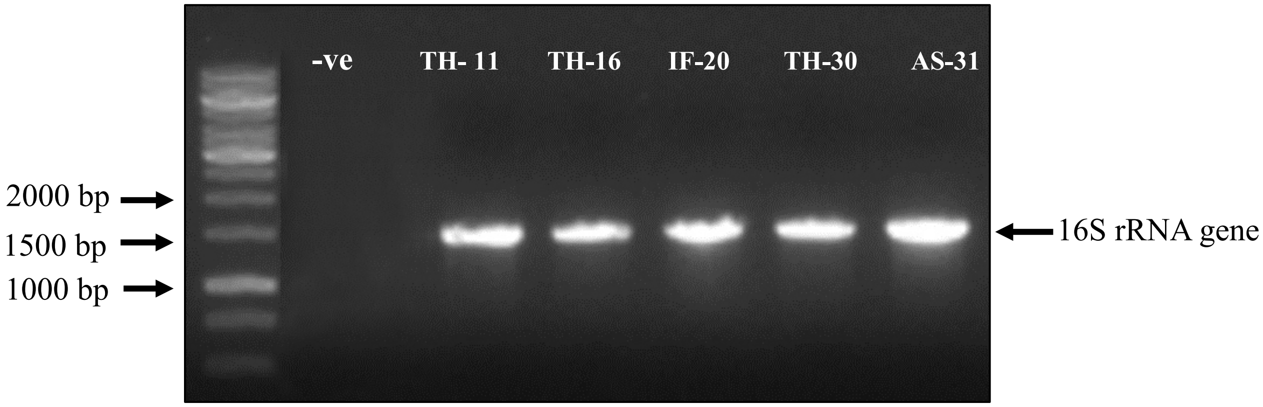

Fig. S14: Agarose gel (1%) showing 16S rRNA genes amplified by PCR with universal primers from the genomic DNA extracted from the E3-transforming strains the strains TH-11, TH-16, IF-20, TH-30, and AS-31. -ve: a negative (no-template) control.

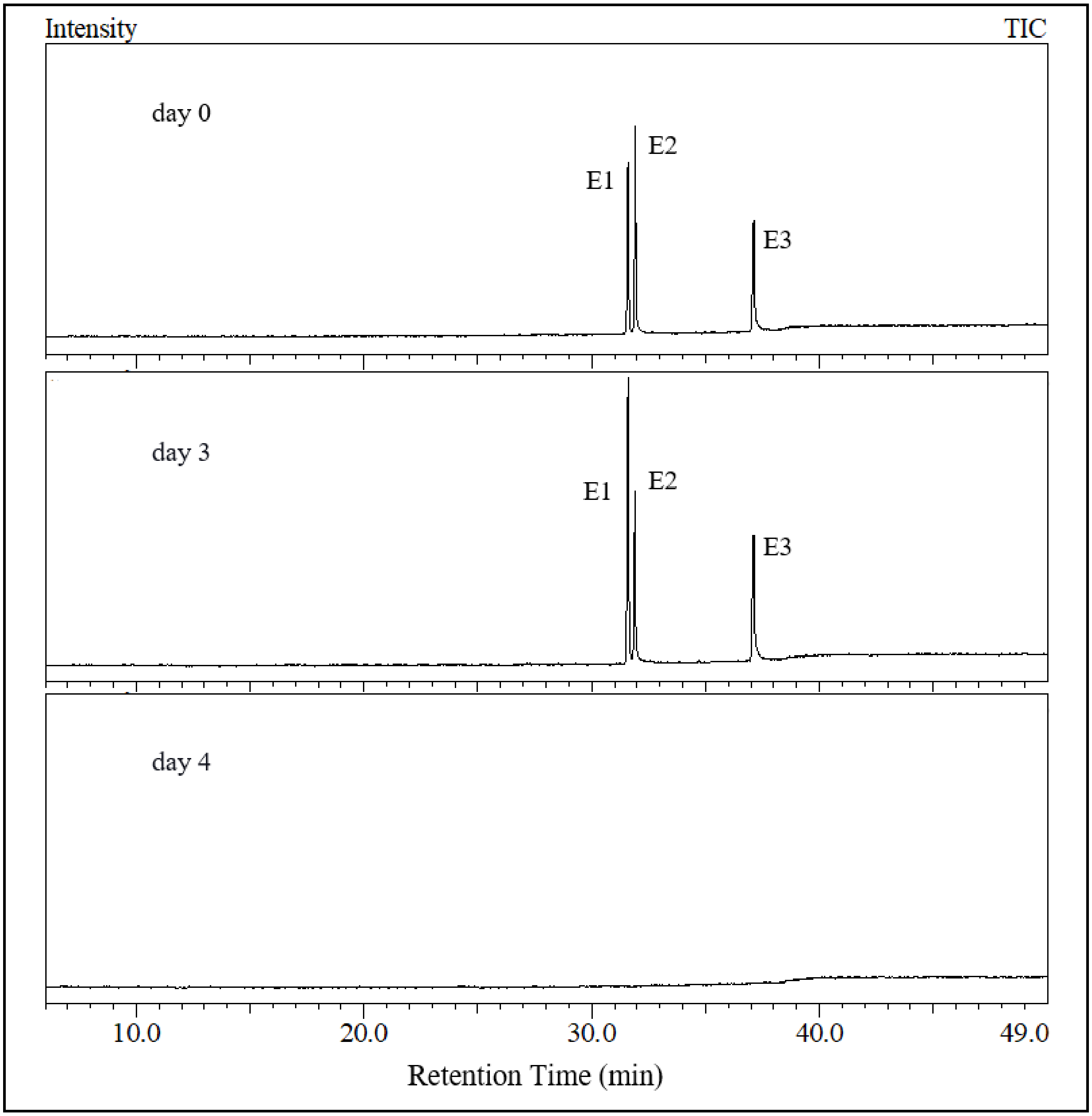

Fig. S15: Total ion chromatograms from GC-MS analysis of cultures and comparison of the cultures at various time points of the IF-consortium in CDM containing a mixture of three estrogens (0.1 mM each), which were added from DMSO stock solutions.

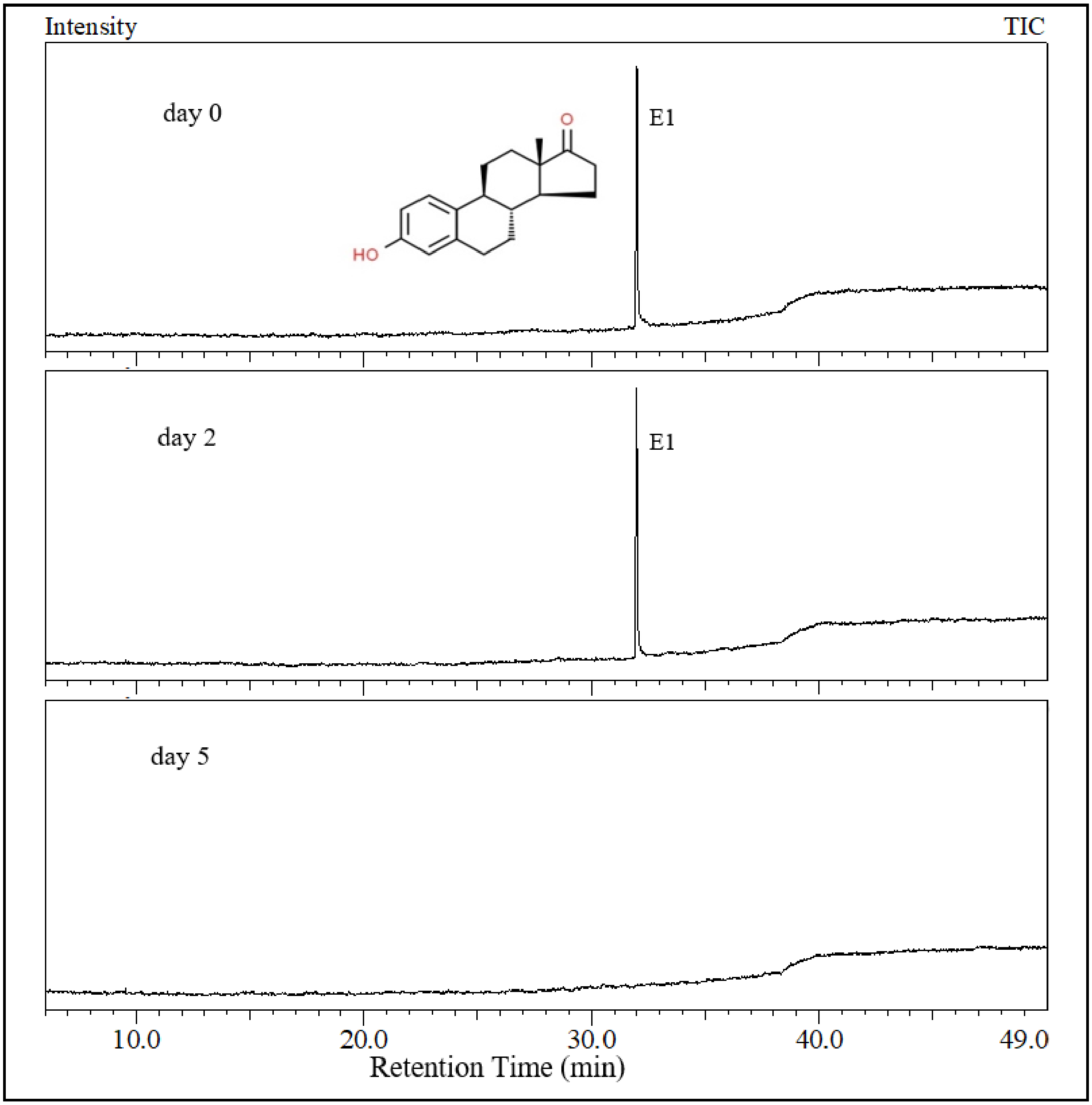

Fig. S16: Total ion chromatograms from GC-MS analysis of cultures of the IF-consortium grown in CDM containing E1 (0.1 mM) as a carbon source. E1 was added from DMSO stock solutions.

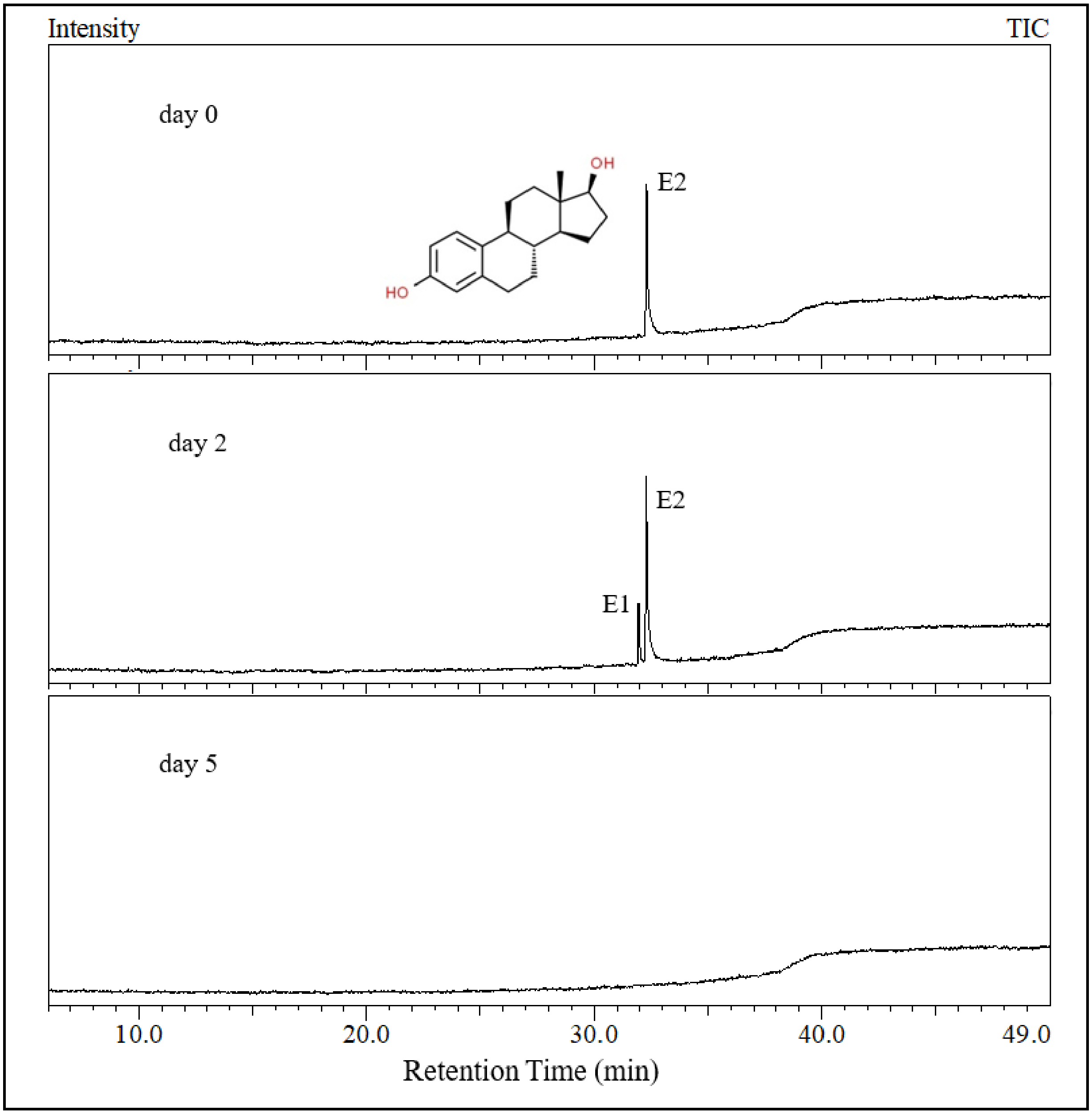

Fig. S17: Total ion chromatograms from GC-MS analysis of cultures of the IF-consortium grown in CDM containing E2 (0.1 mM) as a carbon source. E2 were added from DMSO stock solutions.

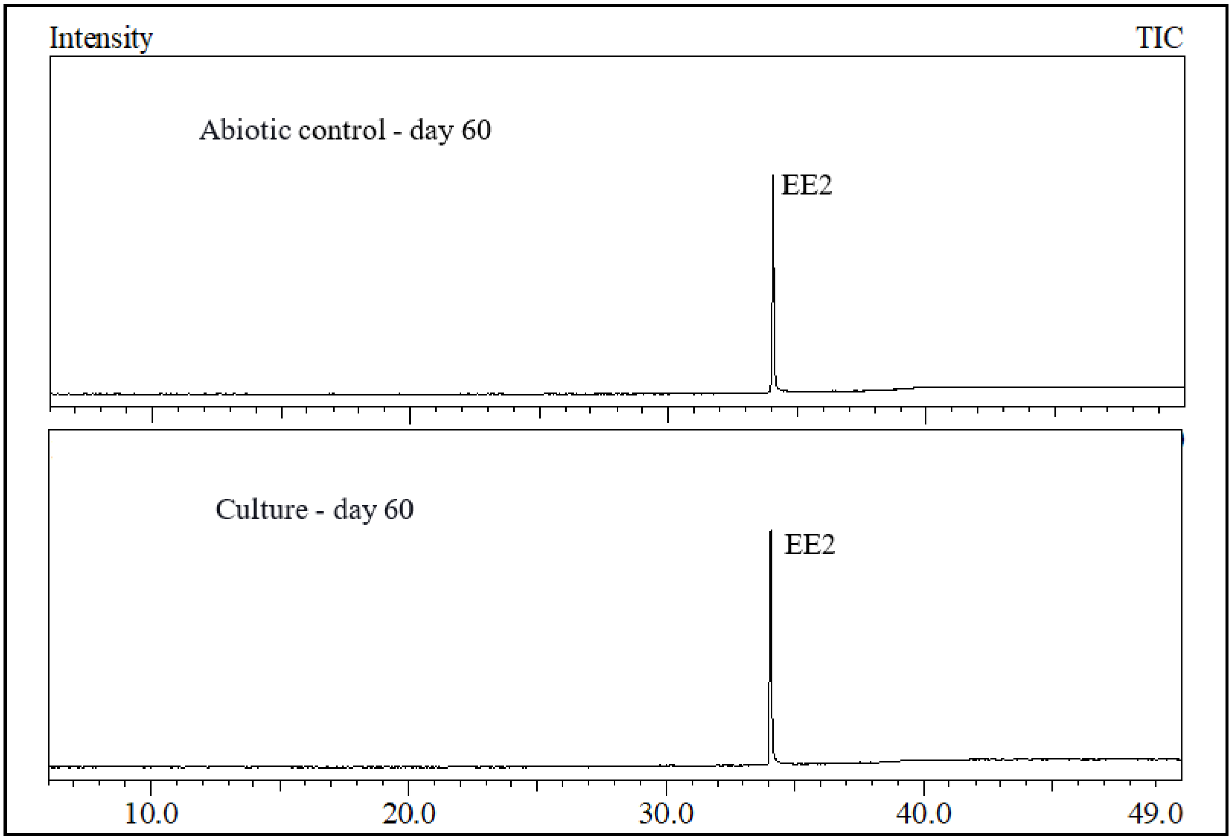

Fig. S18: Total ion chromatograms from GC-MS analysis of cultures of the IF-consortium grown in CDM containing EE2 (0.1 mM) as a carbon source. EE2 was added from a DMSO stock solution.

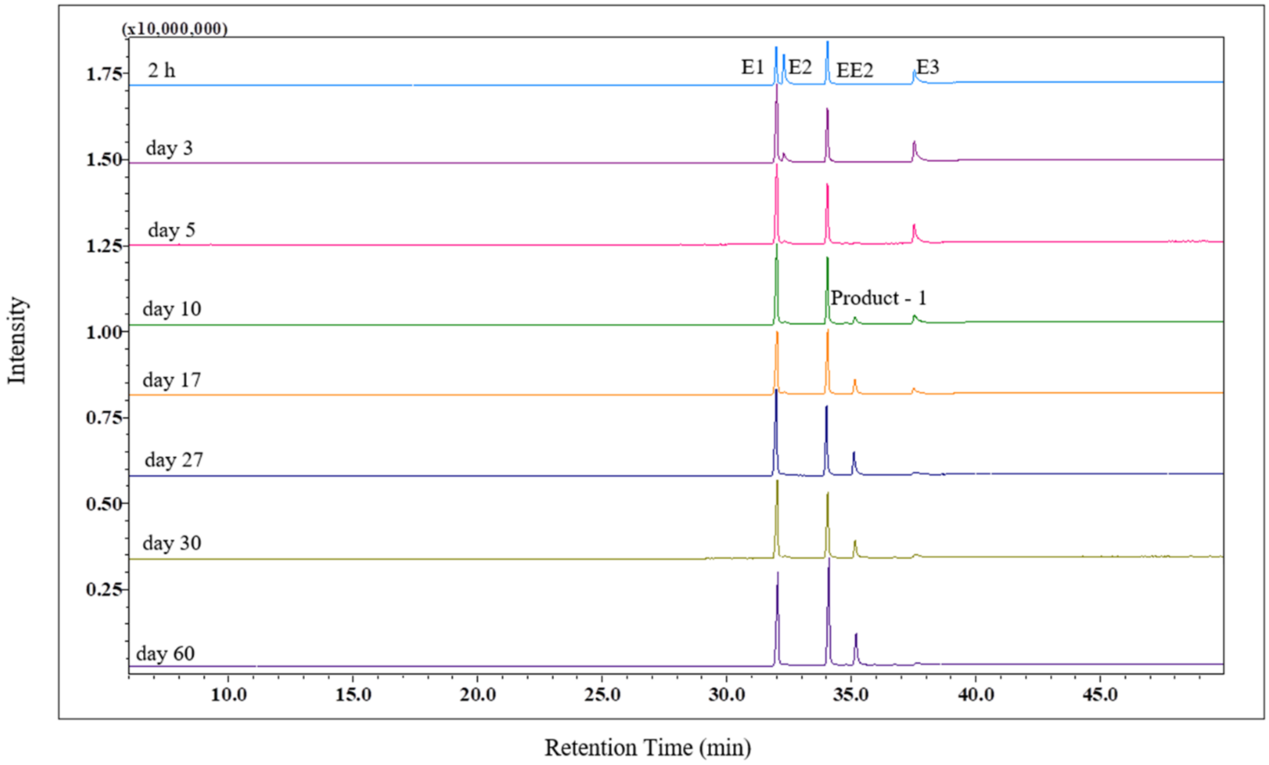

Fig. S19: Total ion chromatograms from GC-MS analysis of cultures of the IF-consortium in CDM containing a mixture of four estrogens (0.1 mM each), which were added from DMSO stock solutions.

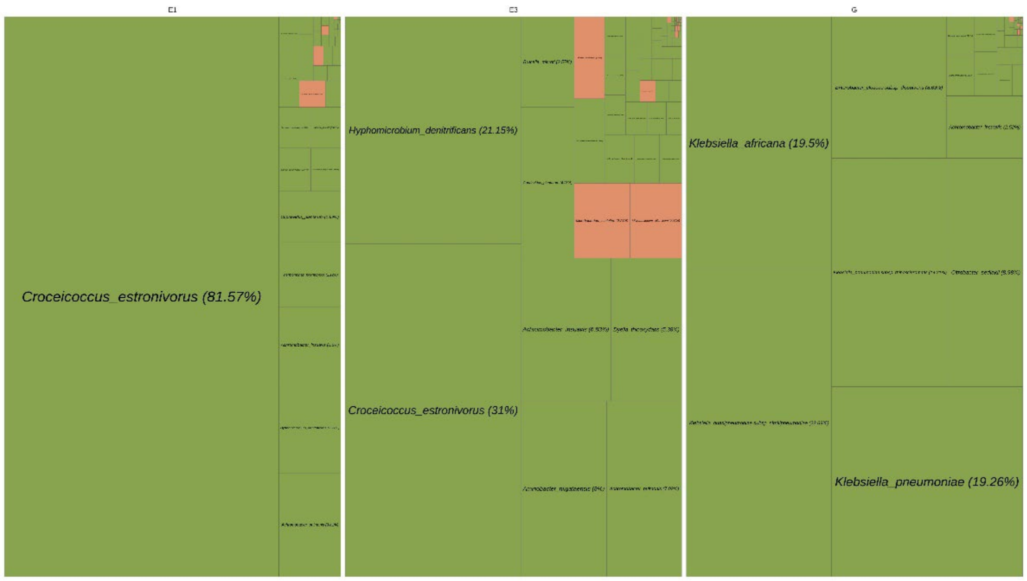

Fig. S20: Species relative abundance dendrogram for the estrone (E1), glucose (G), and estriol (E3) cultures. Green (Pseudomonadota), Pink (Actinomycetota).

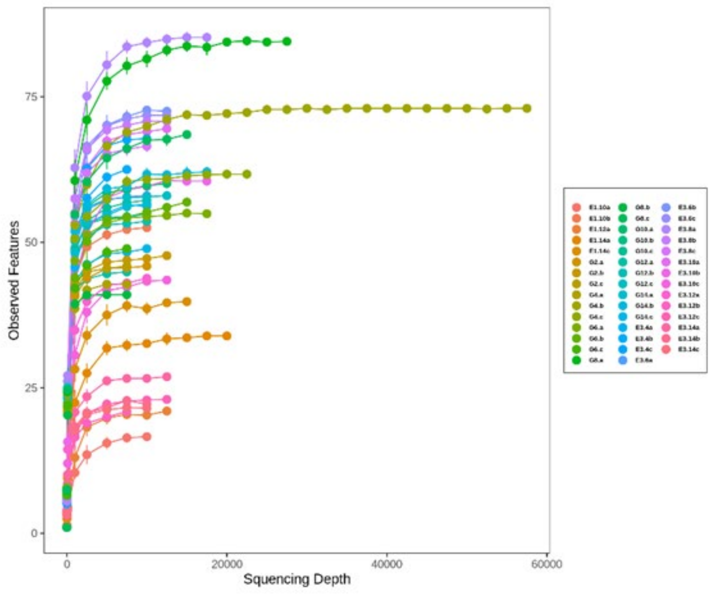

Fig. S21: Rarefaction curve showing ASVs against the sequencing depth.

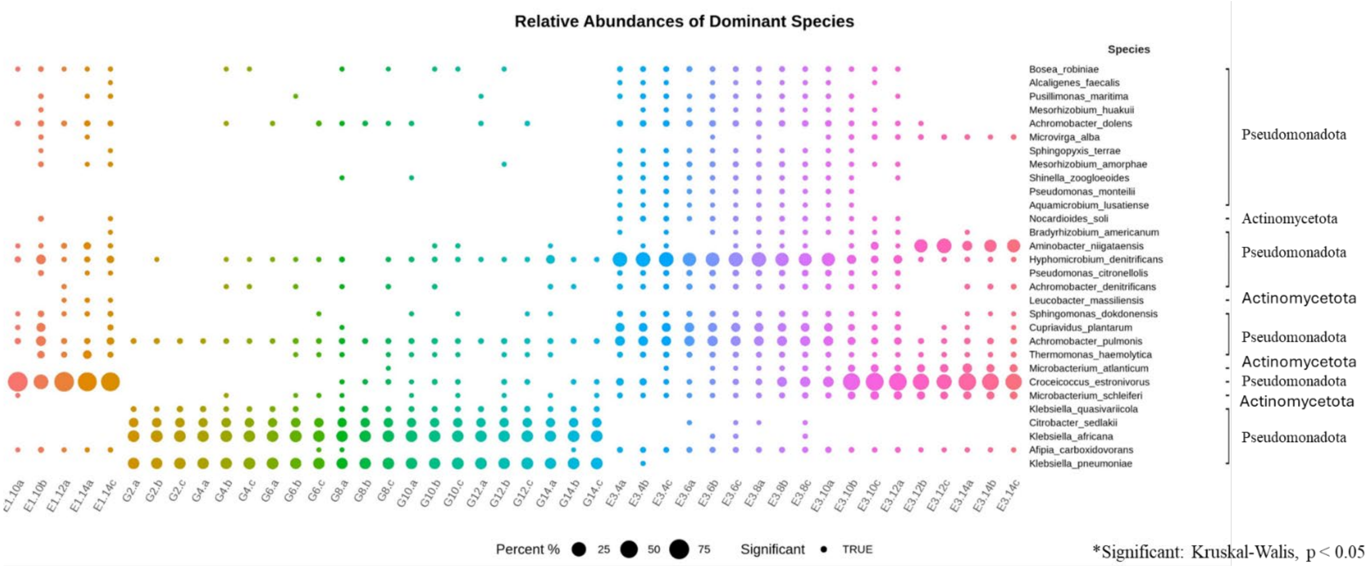

Fig. S22: Dominant species bubble chart showing abundant species with abundance greater than 1% in at least one sample.

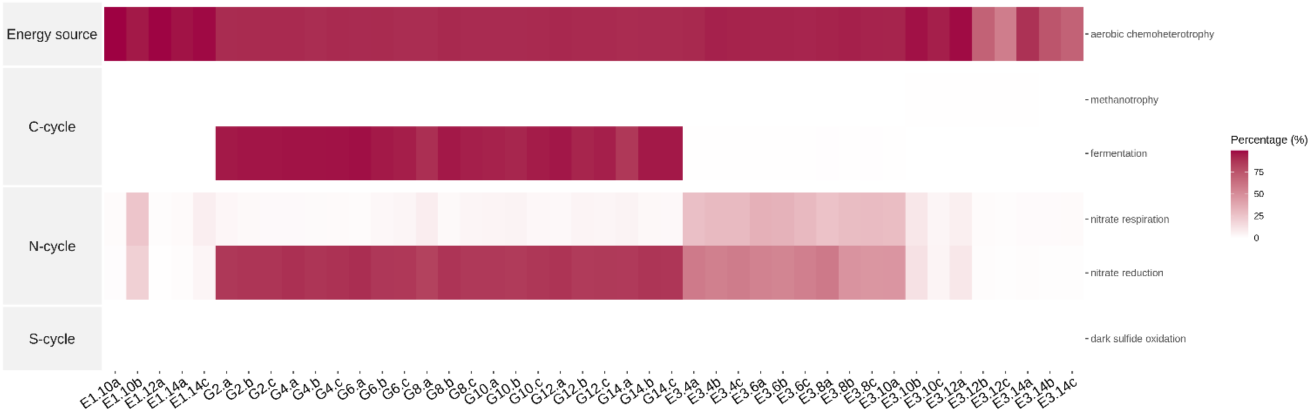

Fig. S23: Major functional differences between the communities identified in the samples collected from the glucose (G), E1 and E3 cultures across individual samples analyzed at different time intervals. Numbers of the sample labels (X-axis) indicate the incubation period in days and the letters designate the biological replicate. FAPROTAX function classifications are displayed on the Y-axis and samples on the X-axis.

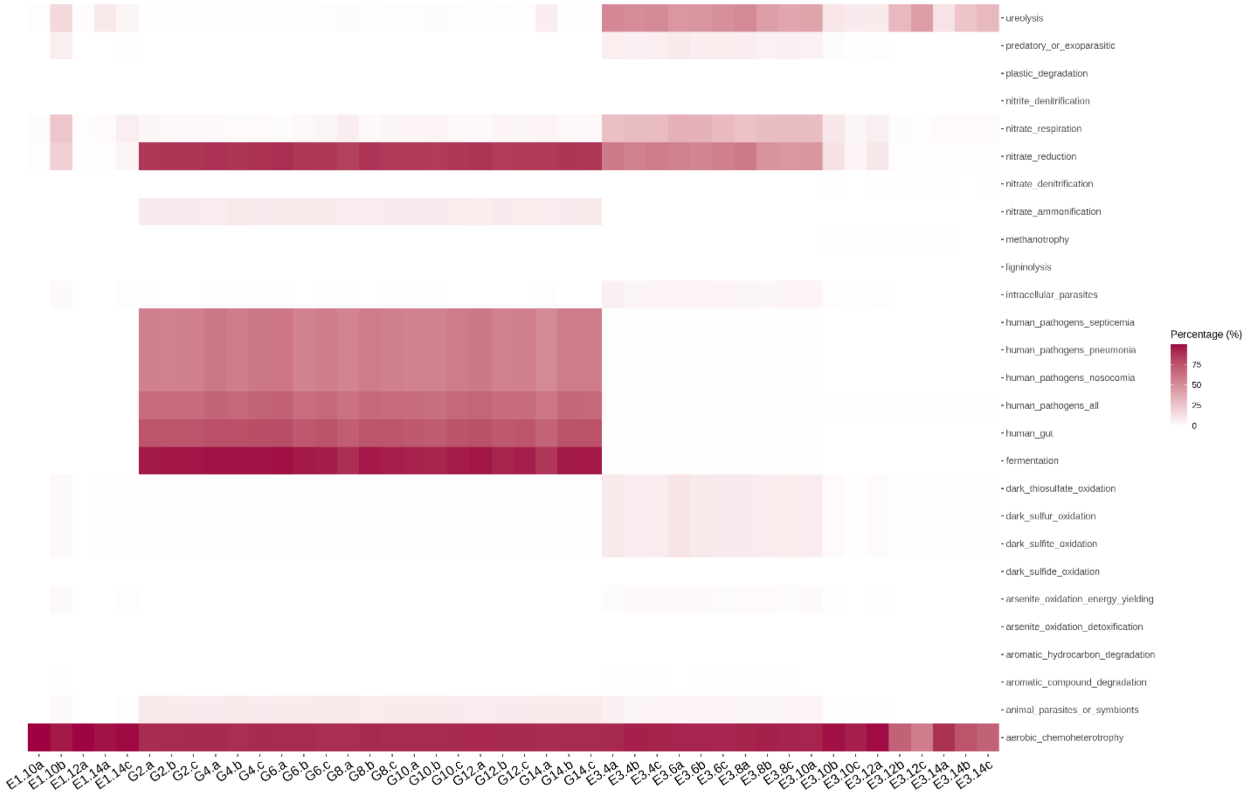

Fig. S24: Key metabolic variations between the microbial communities detected in the glucose (G), E1 and E3 cultures and across individual samples analyzed at different time intervals. Numbers of the sample labels (X-axis) indicate the incubation period in days and the letters designate the biological replicate.

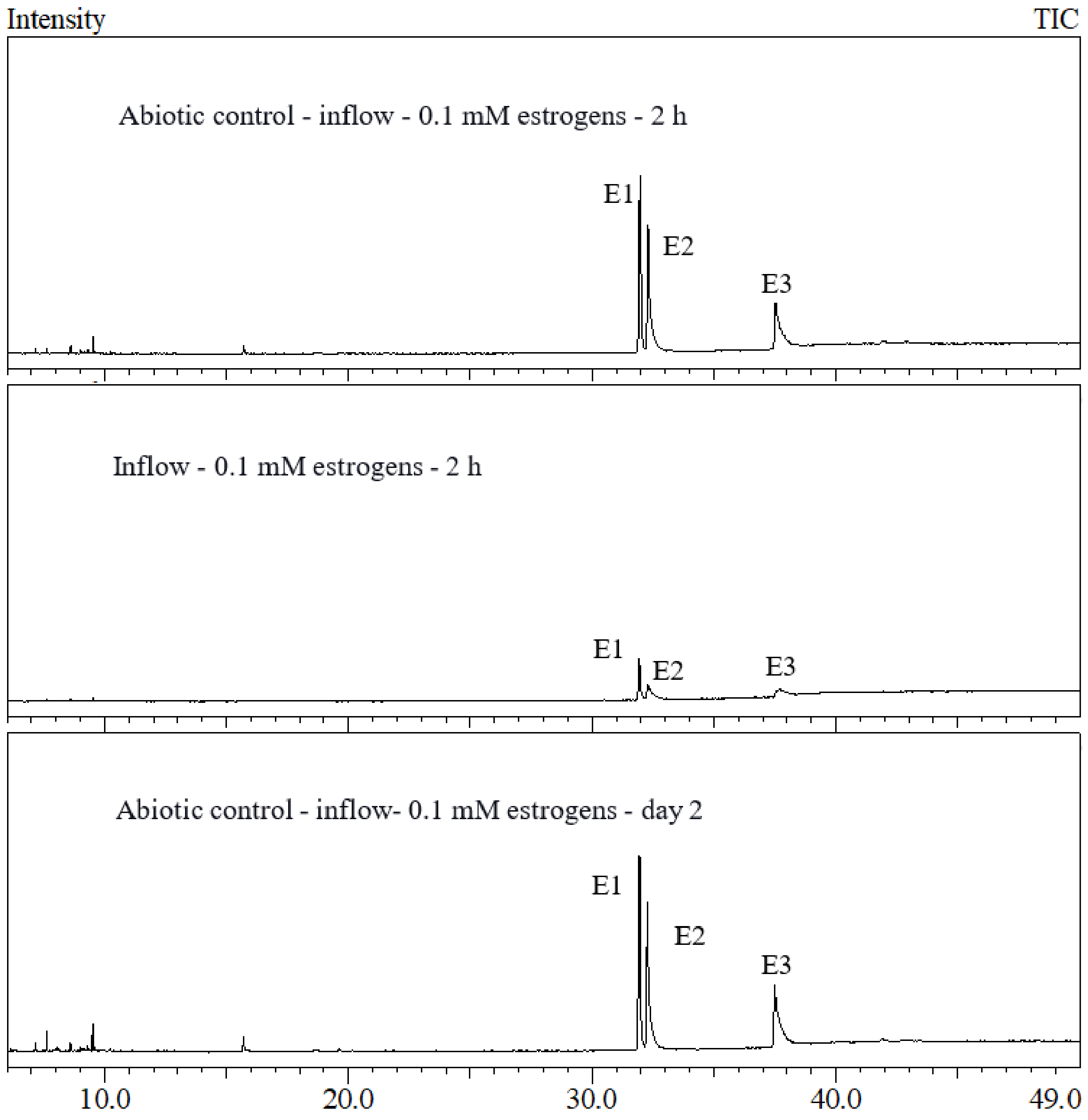

Fig. S25: Legend on next page

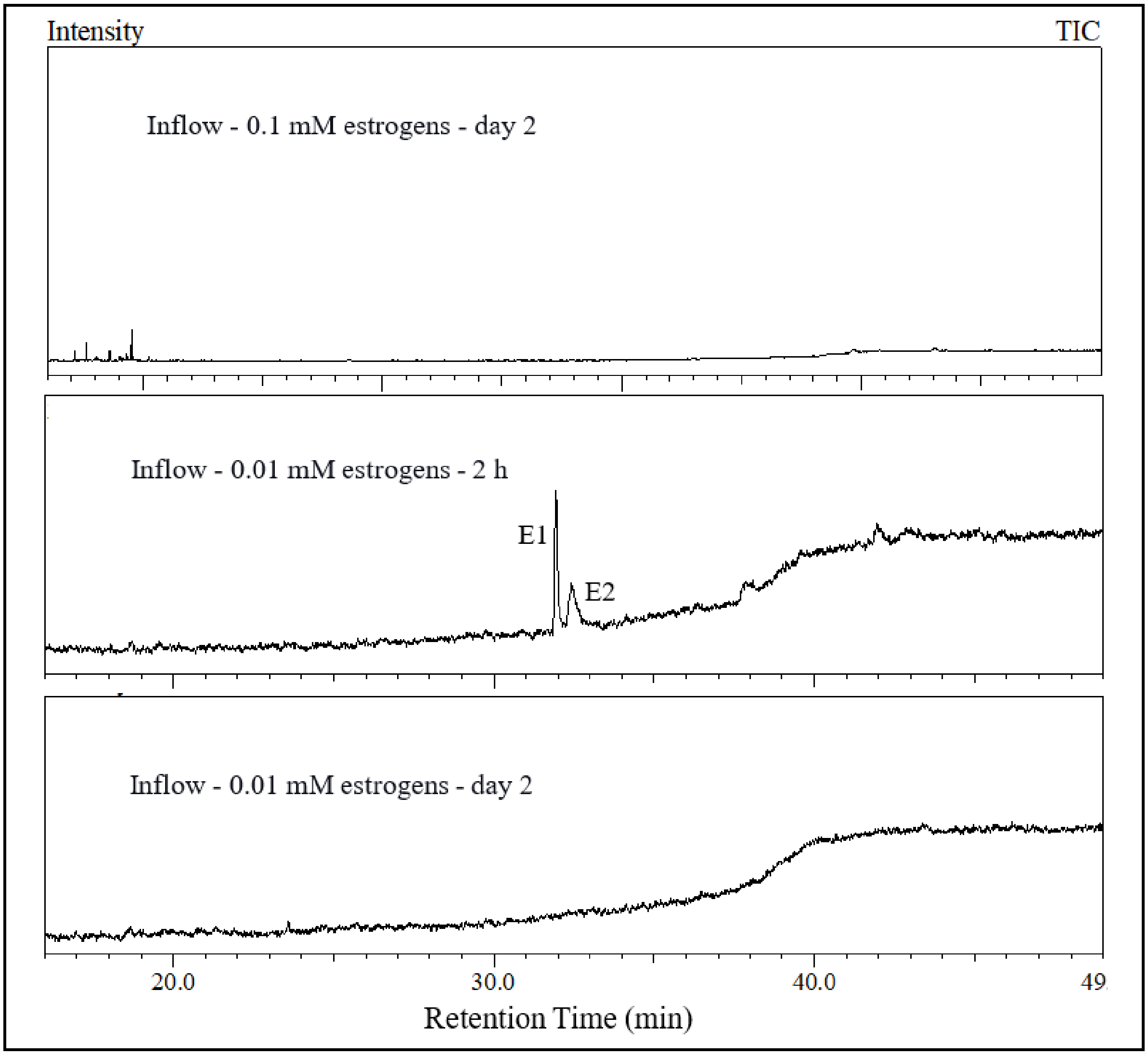

Fig. S25: Total ion chromatograms from GC-MS analysis of the inflow microcosms. Each microcosm contained 500 mL of freshly collected inflow wastewater in 2 L flasks and spiked with a mixture of three estrogens E1, E2, and E3 at two concentrations (0.1 mM or 0.01 mM each) and added from DMSO stock solutions.

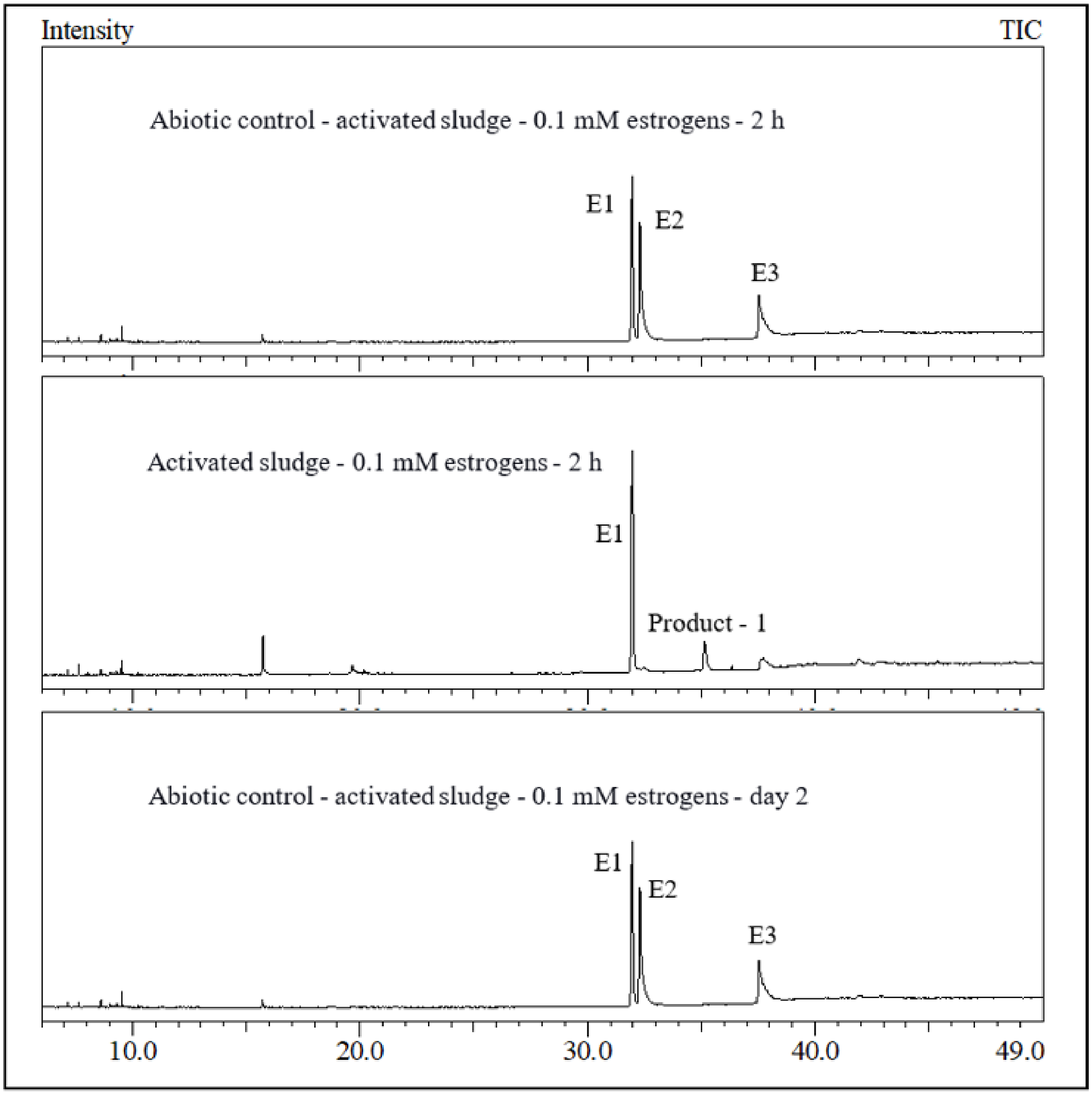

Fig. S26: Legend on next page

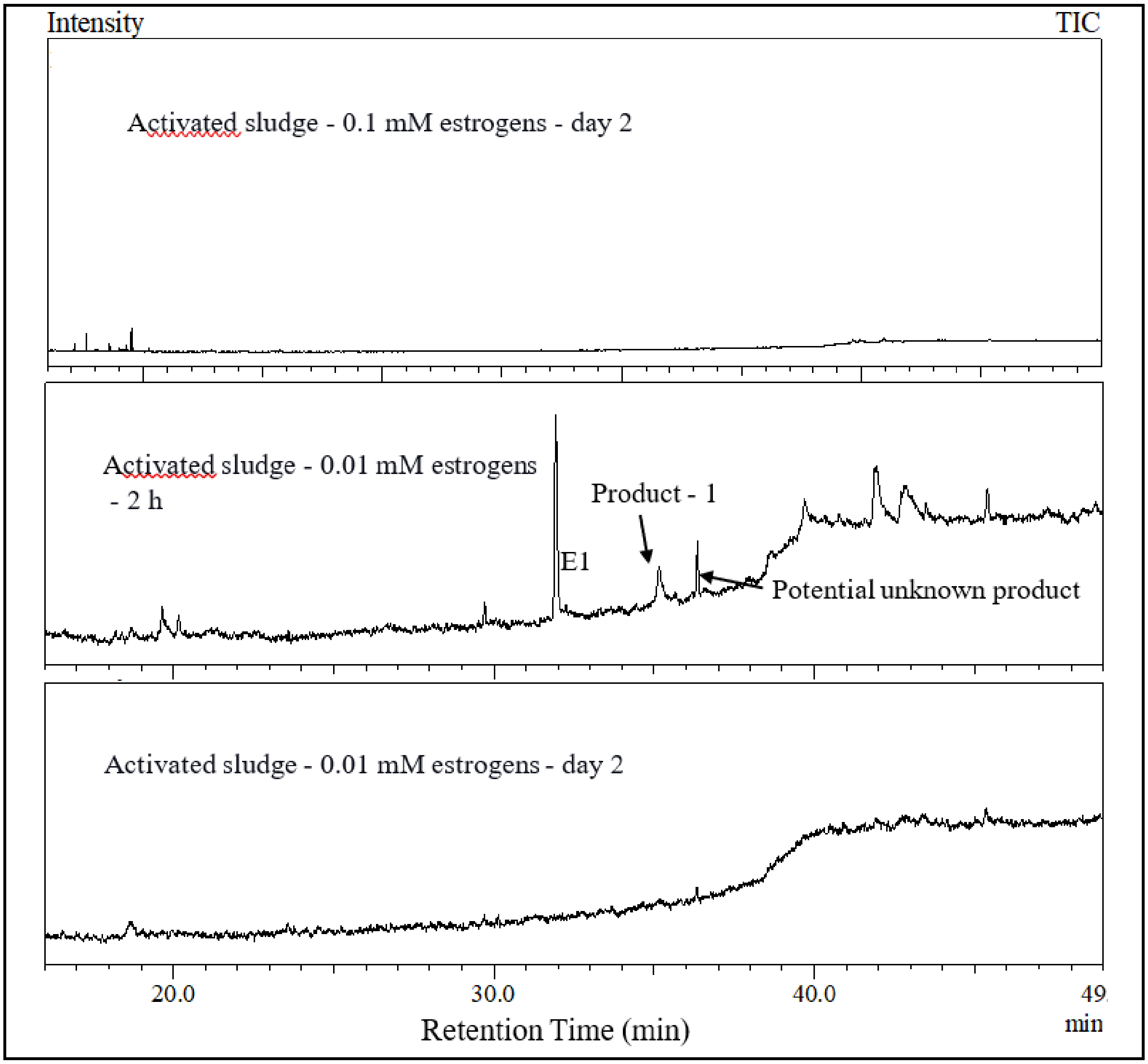

Fig. S26: Total ion chromatograms from GC-MS analysis of the activated sludge microcosms. Each microcosm contained 500 mL of freshly collected activated sludge in 2 L flasks and spiked with a mixture of three estrogens E1, E2, and E3 at two concentrations (0.1 mM or 0.01 mM each) and added from DMSO stock solutions.

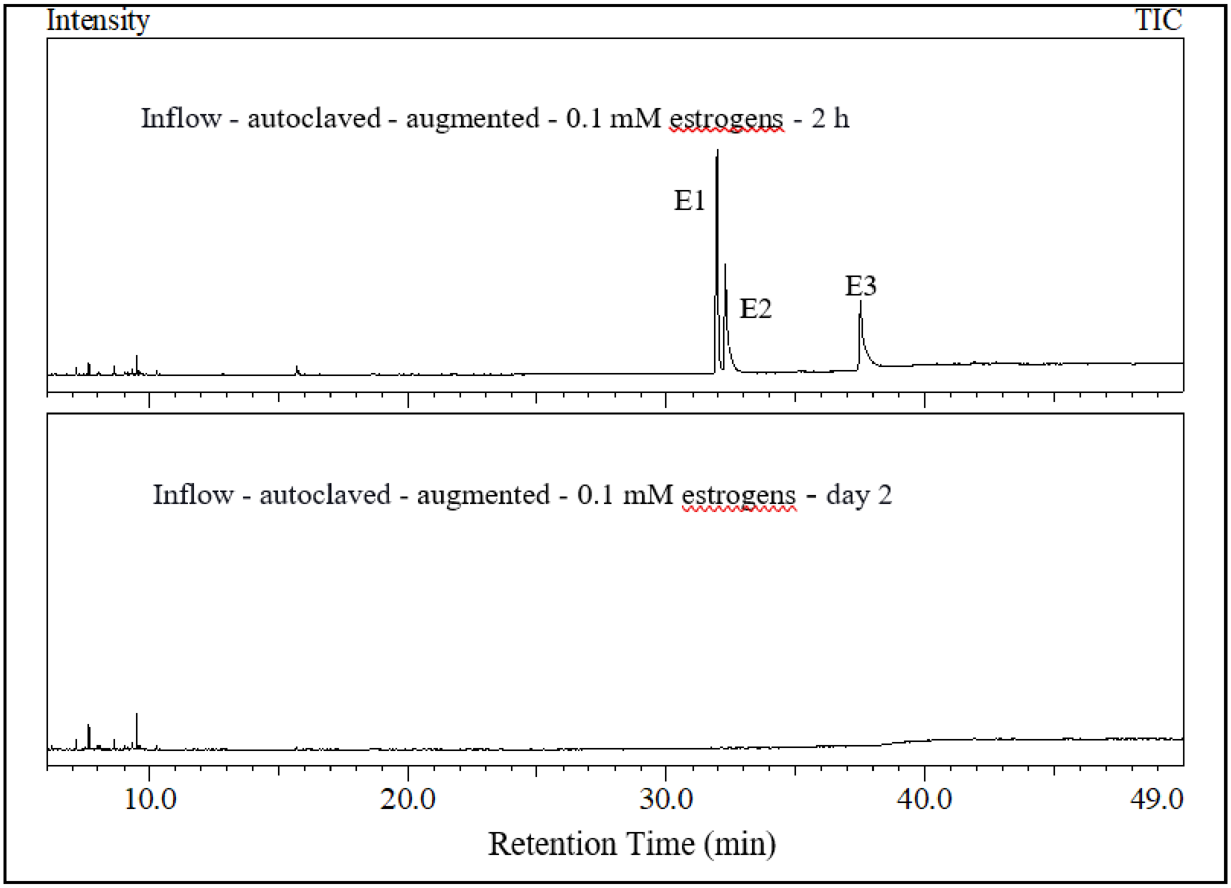

Fig. S27: Total ion chromatograms from GC-MS analysis of the inflow microcosms. Each microcosm contained 500 mL of freshly collected and autoclaved inflow wastewater in 2 L flasks and spiked with a mixture of three estrogens E1, E2, and E3 (0.1 mM each) added from DMSO stock solutions. The microcosms were inoculated with the inflow-consortium.

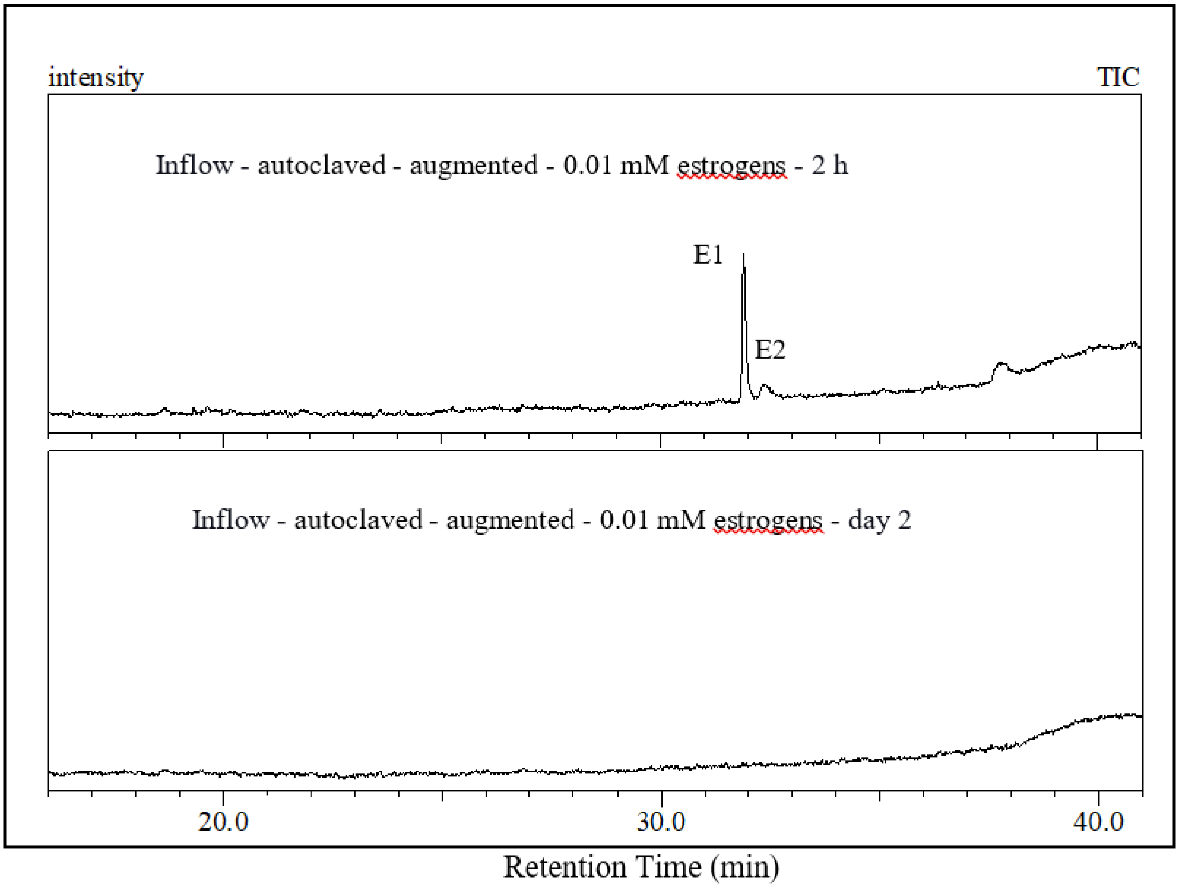

Fig. S28: Total ion chromatograms from GC-MS analysis of the inflow microcosms. Each microcosm contained 500 mL of freshly collected and autoclaved inflow wastewater in 2 L flasks and spiked with a mixture of three estrogens E1, E2, and E3 (0.01 mM each) added from DMSO stock solutions. The microcosms were inoculated with the inflow-consortium.

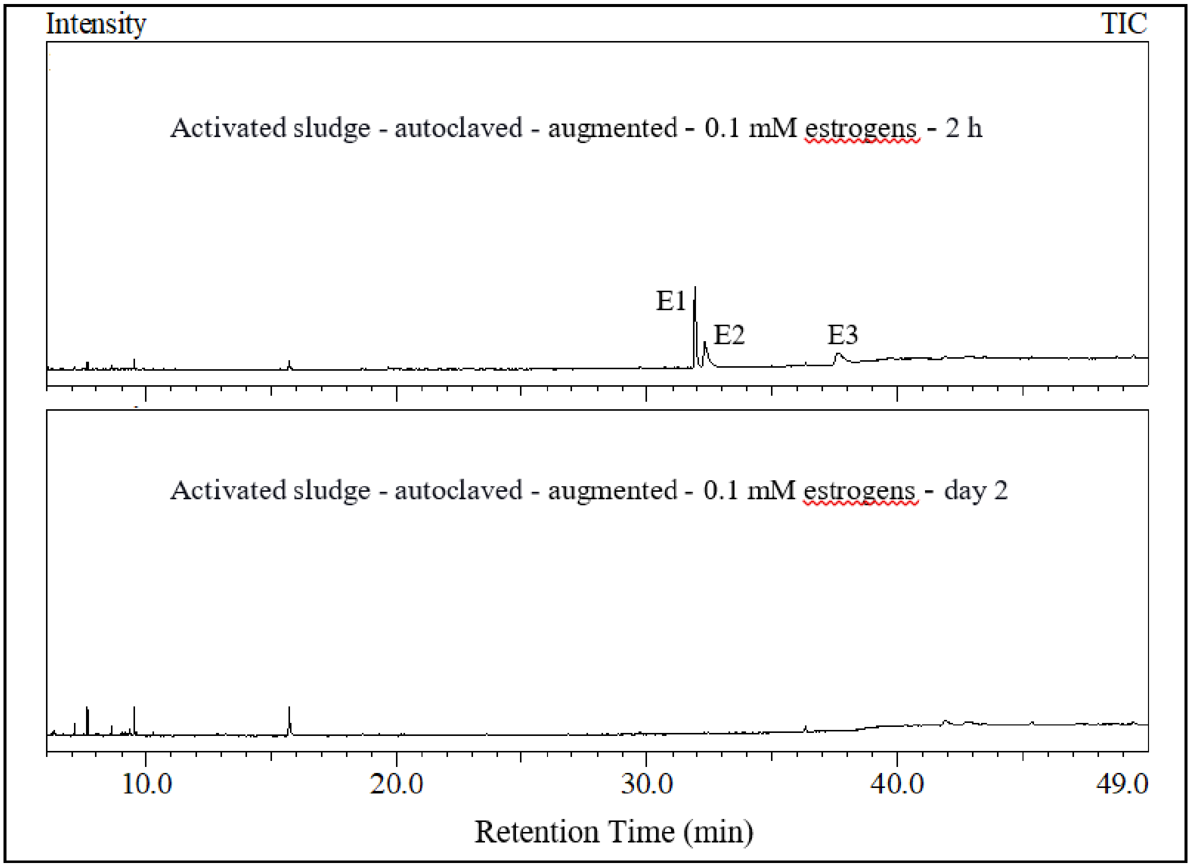

Fig. S29: Total ion chromatograms from GC-MS analysis of the activated sludge microcosms. Each microcosm contained 500 mL of freshly collected and autoclaved activated sludge in 2 L flasks and spiked with a mixture of three estrogens E1, E2, and E3 (0.1 mM each) added from DMSO stock solutions. The microcosms were inoculated with the inflow-consortium.

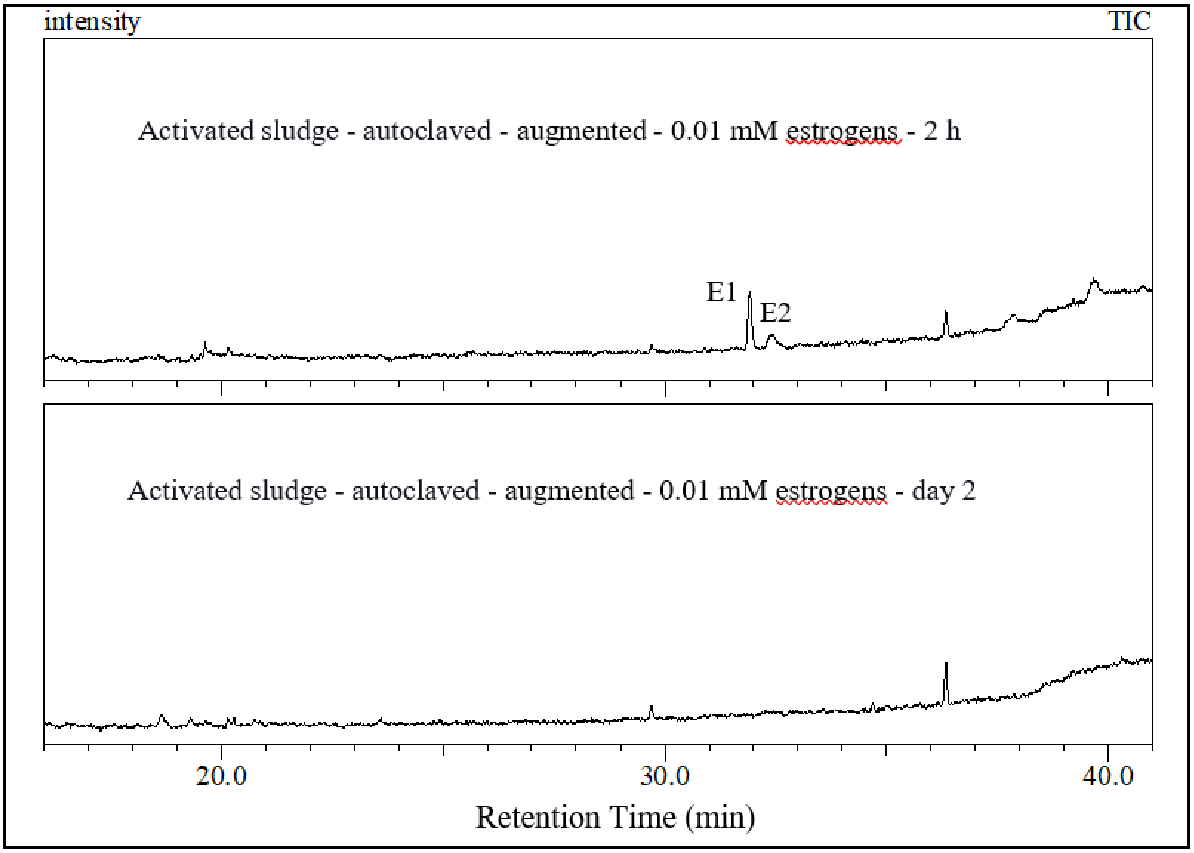

Fig. S30: Total ion chromatograms from GC-MS analysis of the activated sludge microcosms. Each microcosm contained 500 mL of freshly collected and autoclaved activated sludge in 2 L flasks and spiked with a mixture of three estrogens E1, E2, and E3 (0.01 mM each) added from DMSO stock solutions. The microcosms were inoculated with the inflow consortium.

A

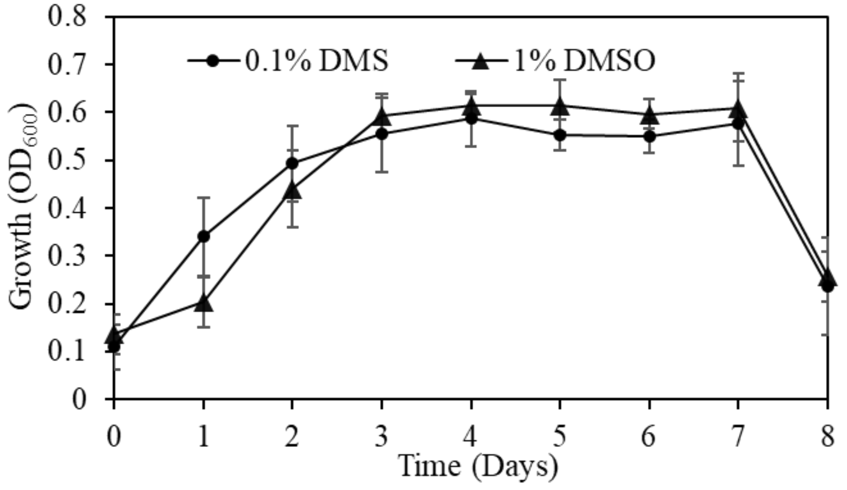

B

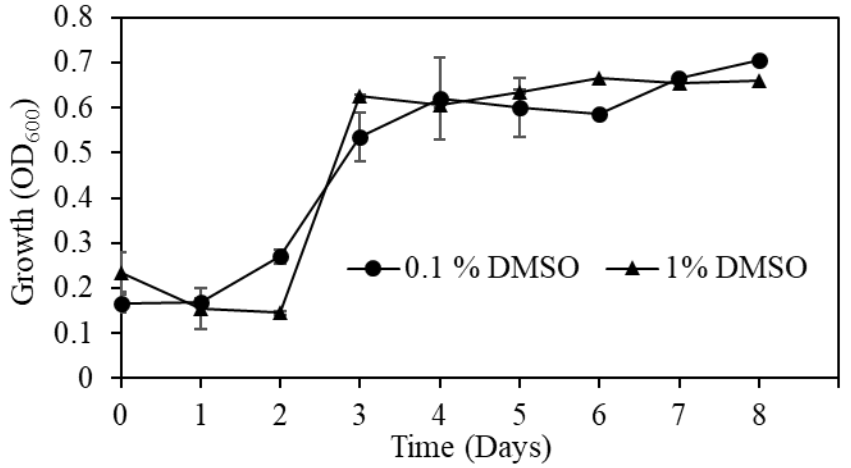

Fig. S31: A. Growth profiles of the IF-consortium on 0.1% and 1.0 % DMSO (v/v) as the sole carbon source. The inoculum was prepared from precultures grown in the presence of 0.1 mM E3. B. Growth profiles of the IF-consortium in CDM cultures containing 0.1 mM E3 and inoculated from precultures grown on either 0.1% or 1.0 % DMSO as the sole carbon source. Data presented as mean of biological triplicates and error bars represent standard error.

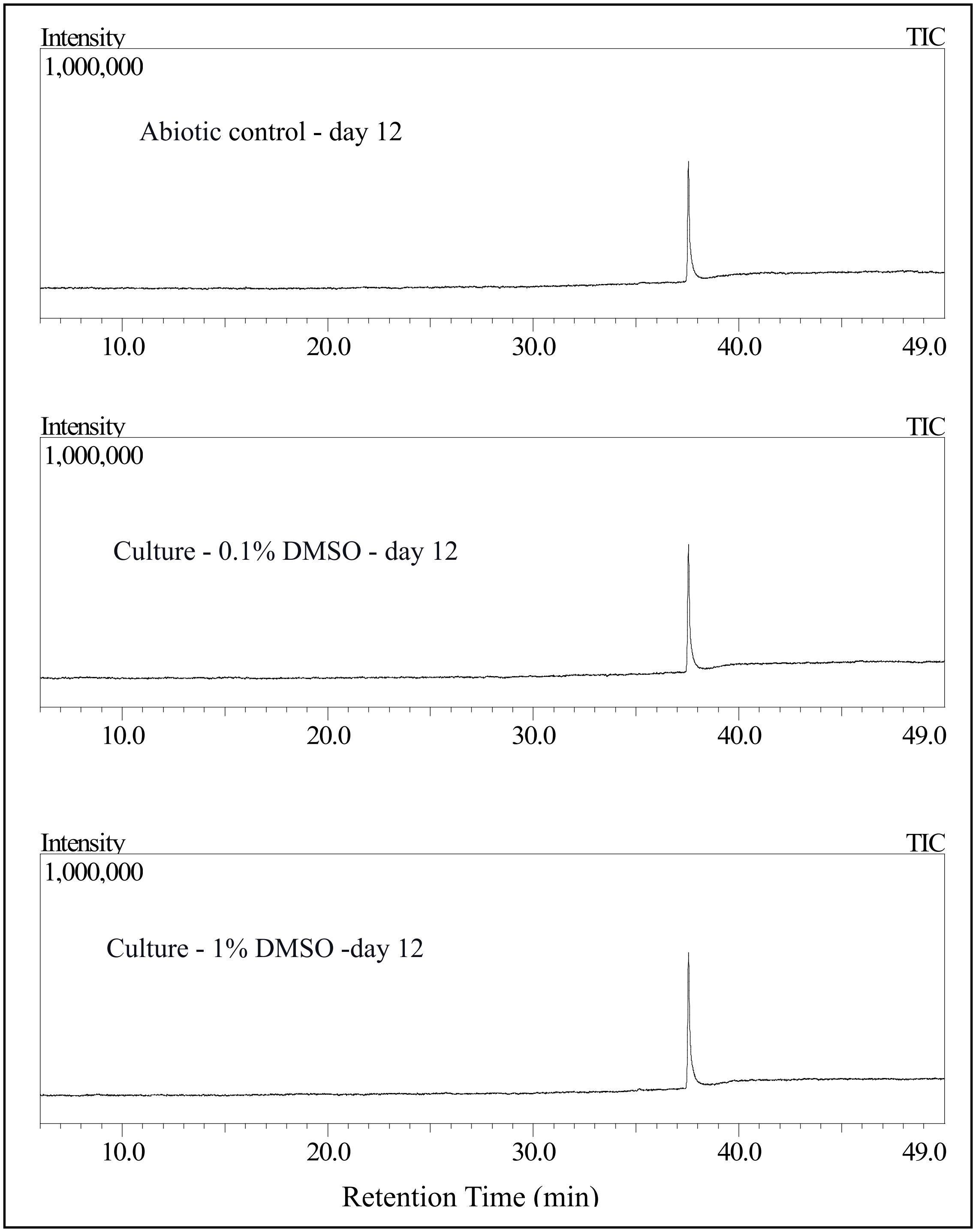

Fig. S32: Total ion chromatogram from GC-MS analysis of E3 (0.1 mM) cultures of the inflow consortium. The inoculum of these cultures was from precultures grown on either 0.1 or 1.0 % DMSO as the sole carbon source. Cultures samples were retrieved after 12 days of incubation, extracted with ethylacetate and analyzed by GC-MS. The peak shown represents E3.

Table S1: Concentrations (mM) of E3 and E1 and their degradation metabolites identified in cultures of the IF-consortium*.

	Days	E1 Culture	E1 (abiotic control)	E3 Culture	E3 (abiotic control)
**E3**					
	0	ND	ND	0.99 ± 0.03	1.03 ± 0.04
	2	ND	ND	0.98 ± 0.04	0.98 ± 0.02
	4	ND	ND	1.02 ± 0.02	0.99 ± 0.03
	6	ND	ND	0.99 ± 0.02	1.02 ± 0.05
	8	ND	ND	0.91 ± 0.01	0.96 ± 0.01
	10	ND	ND	0.83 ± 0.02	0.99 ± 0.04
	12	ND	ND	0.67 ± 0.02	0.95 ± 0.03
	14	ND	ND	0.23 ± 0.04	0.98 ± 0.05
**E1**		E1 Culture	E1 (abiotic control)	E3 Culture	E3 (abiotic control)
	0	1.00 ± 0.02	0.97 ± 0.05	0.00 ± 0.00	ND
	2	0.97 ± 0.03	1.02 ± 0.04	0.00 ± 0.00	ND
	4	0.94 ± 0.04	0.99 ± 0.03	0.00 ± 0.00	ND
	6	0.91 ± 0.02	1.01 ± 0.02	0.01 ± 0.00	ND
	8	0.88 ± 0.03	0.99 ± 0.03	0.05 ± 0.02	ND
	10	0.76 ± 0.02	0.97 ± 0.02	0.14 ± 0.02	ND
	12	0.61 ± 0.04	0.98 ± 0.03	0.28 ± 0.04	ND
	14	0.48 ± 0.01	1.00 ± 0.02	0.61 ± 0.03	ND
**4-hydroxyestrone**		E1 Culture	E1 (abiotic control)	E3 Culture	E3 (abiotic control)
	0	0.00 ± 0.00	ND	0.00 ± 0.00	ND
	2	0.00 ± 0.00	ND	0.00 ± 0.00	ND
	4	0.01 ± 0.00	ND	0.00 ± 0.00	ND
	6	0.02 ± 0.00	ND	0.00 ± 0.00	ND
	8	0.03 ± 0.01	ND	0.00 ± 0.00	ND
	10	0.05 ± 0.02	ND	0.01 ± 0.00	ND
	12	0.08 ± 0.03	ND	0.02 ± 0.00	ND
	14	0.11 ± 0.03	ND	0.04 ± 0.01	ND
**PEA**		E1 Culture	E1 (abiotic control)	E3 Culture	E3 (abiotic control)
	0	0.00 ± 0.00	ND	0.00 ± 0.00	ND
	2	0.00 ± 0.00	ND	0.00 ± 0.00	ND
	4	0.00 ± 0.00	ND	0.00 ± 0.00	ND
	6	0.01 ± 0.00	ND	0.00 ± 0.00	ND
	8	0.01 ± 0.00	ND	0.00 ± 0.00	ND
	10	0.02 ± 0.00	ND	0.01 ± 0.00	ND
	12	0.02 ± 0.01	ND	0.01 ± 0.00	ND
	14	0.05 ± 0.02	ND	0.02 ± 0.00	ND

**HIP**		E1 Culture	E1 (abiotic control)	E3 Culture	E3 (abiotic control)
	0	0.00 ± 0.00	ND	0.00 ± 0.00	ND
	2	0.00 ± 0.00	ND	0.00 ± 0.00	ND
	4	0.00 ± 0.00	ND	0.00 ± 0.00	ND
	6	0.00 ± 0.00	ND	0.00 ± 0.00	ND
	8	0.01 ± 0.00	ND	0.00 ± 0.00	ND
	10	0.02 ± 0.00	ND	0.00 ± 0.00	ND
	12	0.01 ± 0.00	ND	0.01 ± 0.00	ND
	14	0.03 ± 0.01	ND	0.01 ± 0.00	ND

*ND: not detected
